# The Effect of Vitamins During Pregnancy on Pregnancy and Assisted Reproduction Outcomes: An Umbrella Review

**DOI:** 10.3390/jcm15218434

**Published:** 2026-10-29

**Authors:** Yuzhu Zhang, Huimin Liu, Lang Qin, Rui Gao

**Affiliations:** 1 Reproductive Medical Center, Department of Obstetrics and Gynecology, West China Second University Hospital, Sichuan University, No. 1416, Section 1, Chenglong Avenue, Jinjiang District, Chengdu 610041, China; 2 Key Laboratory of Birth Defects and Related Diseases of Women and Children, Ministry of Education, West China Second University Hospital, Sichuan University, Chengdu 610041, China

**Keywords:** vitamin, pregnancy, maternal outcomes, fetal outcomes, assisted reproductive outcomes

## Abstract

**Background:** Vitamin deficiency is associated with multiple adverse maternal and fetal outcomes. **Objective:** To summarize systematic reviews and meta-analyses, and to evaluate the impact of vitamin deficiency and vitamin supplementation during pregnancy on maternal and fetal outcomes, including outcomes of assisted reproductive technology (ART). **Methods:** Prior to conducting this umbrella review, we registered the protocol in PROSPERO (CRD420251026875). We searched PubMed, Embase and the Cochrane Library for all systematic reviews and meta-analyses concerning vitamin status assessment and supplementation during pregnancy up to April 2025, and extracted all relevant pregnancy outcomes associated with prenatal vitamins. Screening, data extraction, and quality assessment (AMSTAR 2) were completed by two independent reviewers. Data were quantitatively synthesized, and odds ratios (OR) with 95% confidence intervals (95%CI) were mainly reported. **Results:** A total of 33 eligible systematic reviews and meta-analyses covering 679 primary studies were included, which explored 29 maternal–fetal outcomes related to six vitamins from the perspectives of vitamin insufficiency/deficiency and supplementation. Vitamin B12, folate, vitamin C and D deficiencies were linked to higher risks of gestational diabetes mellitus (GDM), pre-eclampsia, low birth weight, recurrent spontaneous abortion (RSA), preterm birth and other adverse outcomes. Low vitamin D also correlated with poorer assisted reproductive outcomes including reduced clinical pregnancy and live birth rates. Appropriate supplementation of folate, vitamin C and D may lower the risks of megaloblastic anemia, premature rupture of membranes, GDM and stillbirth, and improved neonatal growth. Vitamin A exerted no notable influence on pregnancy outcomes. **Conclusions:** Insufficiency or deficiency in vitamin B12, folic acid, vitamin C, and vitamin D is more likely to result in adverse pregnancy outcomes. Supplementation with folic acid, vitamin C and vitamin D during pregnancy can improve the incidence of maternal and fetal outcomes to some extent.

## 1. Background

Micronutrients are essential for optimal pregnancy outcomes and the normal metabolic activities that support fetal growth and function. During pregnancy, maternal nutritional status not only directly impacts one’s own health but also regulates fetal growth and development through the placental barrier, having a profound effect on the short- and long-term outcomes for both mother and infant. Globally, adverse pregnancy outcomes such as preterm birth, low birth weight, pre-eclampsia and gestational diabetes mellitus (GDM) remain major public health issues threatening maternal and infant health. Nutritional intervention, as a modifiable primary prevention strategy, holds significant clinical importance in improving pregnancy outcomes and reducing the risk of maternal and infant morbidity. Furthermore, as vitamins are key micronutrients that are essential for maintaining the body’s normal physiological functions, supplementation interventions during pregnancy have become a research hotspot in the field.

Vitamins play multiple physiological roles in metabolic processes during pregnancy. Numerous systematic reviews have examined the effects of vitamin deficiency or insufficiency, as well as supplementation, on maternal and infant outcomes. The evidence regarding folic acid in the prevention of infant neural tube defects has become the gold standard, and standard supplementation of 0.36–4.00 mg/day of folic acid from preconception through early pregnancy can reduce the risk of neural tube defects by 50–70% [1,2]. Vitamin D receptors are widely distributed in tissues such as the placenta and pancreatic islets. Low-serum 25-hydroxyvitamin D levels are considered to be closely associated with recurrent spontaneous abortion [3], GDM [4], pre-eclampsia [5], preterm birth [6], and small for gestational age (SGA) infants [6].

However, there are significant differences in the mechanisms of action and the strength of evidence regarding the effects of different vitamins on pregnancy outcomes. Although certain vitamin deficiencies, such as folate deficiency, are known to adversely affect pregnancy or assisted reproduction outcomes, the existing evidence remains inconsistent and inconclusive. Conflicting findings across primary studies and systematic reviews hinder the development of unified, reliable, and actionable clinical guidelines for prenatal vitamin intervention. Given the lack of comprehensive evidence synthesis to clarify the robustness of these associations, this umbrella review aims to systematically integrate published systematic reviews and meta-analyses addressing prenatal vitamin status and supplementation. By summarizing and evaluating associations between vitamin-related indicators and both pregnancy and assisted reproductive outcomes, this study further clarifies the magnitude and reliability of the available evidence. The findings will provide an evidence base for optimizing antenatal nutritional care, developing standardized supplementation strategies, and informing future high-quality research in maternal–fetal nutrition.

## 2. Methods

### 2.1. Protocol and Registration

This umbrella review was reported in accordance with the PRIOR statement and a completed PRISMA 2020 checklist is provided in the Supplementary Materials. Prior to conducting this review, we registered the protocol in PROSPERO with the number CRD420251026875.

### 2.2. Study Retrieval and Selection

Three electronic databases (PubMed, Embase, and the Cochrane Library) were searched from their inception to April 2025 to identify relevant systematic reviews and meta-analyses exploring the effects of vitamin insufficiency/deficiency and supplementation on pregnancy and assisted reproductive technology (ART) outcomes. The complete search strategy is presented in Supplementary Table S1. Initial deduplication was performed using EndNote 20, followed by title/abstract screening. Finally, full-text screening was conducted to determine whether the remaining meta-analyses met the inclusion criteria. Two authors (Y.Z. and H.L.) independently performed the full-text screening. Any disagreements were resolved through discussion. Furthermore, we manually searched the reference lists of the included systematic reviews and meta-analyses, or related reviews, to identify potential meta-analyses for inclusion.

### 2.3. Inclusion and Exclusion Criteria

We employed the PECOS or PICOS (P: patients, E: exposure, I: intervention, C: comparison, O: outcome, S: study design) framework to determine the inclusion criteria. The criteria were as follows: (1) patients: pregnant women; (2) exposure: measured vitamin levels; (3) intervention: vitamin intake levels; (4) comparison: low-dose vitamin supplementation, placebo, no supplementation, or low measured vitamin levels as defined by the authors; (5) outcomes: all pregnancy and fetal outcomes; and (6) study design: meta-analyses of randomized controlled trials or observational studies. The exclusion criteria were as follows: (1) conference abstracts; (2) animal studies; (3) meta-analyses not part of a systematic review; and (4) meta-analyses not published in English.

### 2.4. Data Extraction

We extracted the following information from the included meta-analyses: first author’s name, publication year, type of clinical outcome, study design, number of studies in each meta-analysis, total sample size, and effect sizes (OR: odds ratio, RR: relative risk, MD: mean difference, SMD: standardized mean difference). Two independent reviewers (Y.Z. and H.L.) extracted data from the reviews. If there were discrepancies, any disagreements were resolved through consensus or by consulting a third reviewer (R.G).

Pooled estimates including 95% confidence intervals (CIs) for clinical outcomes were extracted from each included meta-analysis. Evidence of heterogeneity (I^2^ values) was also extracted from the meta-analyses. In addition, where available, publication bias, assessed by Egger’s or Begg’s tests, was also extracted from the included meta-analyses.

### 2.5. Quality Assessment

Two reviewers independently completed the methodological quality assessment of each meta-analysis using the AMSTAR 2 tool. Any disagreements were resolved by consulting a third reviewer (L.Q). Out of the 16 items on the AMSTAR 2 checklist, 7 are considered critical. The tool can categorize the quality of each meta-analysis into four classes: high (no or only one non-critical weakness), moderate (contains multiple non-critical weaknesses), low (contains one critical flaw among: protocol registration, comprehensive literature search, justification for excluding studies, risk of bias assessment for each included study, appropriate meta-analytical methods, consideration of risk of bias when interpreting review results, and assessment of publication bias), and critically low (contains multiple critical flaws). Reviews rated as critically low quality were excluded. Detailed information on the quality assessment can be found in Supplementary Table S2.

### 2.6. Reviews with Overlapping Primary Studies

If a meta-analysis overlaps with multiple similar outcomes, the coverage area (CCA) is calculated to quantify the degree of overlap. (1) A CCA below 10% indicates little or no overlap between studies. In such cases, the original studies should be extracted separately and reanalyzed. (2) A CCA of 10% or higher indicates moderate to high overlap. We referred to an umbrella review published in the BMJ in 2023 to guide the selection of included studies [7]. For meta-analyses published more than 24 months apart, the most recently published one was selected for data extraction and analysis. For studies published within 24 months of each other, the meta-analysis incorporating the largest number of original studies was selected. If the number of studies was equal, the study with the highest AMSTAR-2 score was included. The calculation formula is shown in Equation (1). Detailed information regarding the handling of overlapping studies can be found in Supplementary Table S3.

Equation (1) CCA calculation method.

CCA (%) = N − r/rc − r(1)

N: Number of included publications (sum of checked boxes), r: number of rows (primary studies), c: number of columns (number of systematic reviews).

### 2.7. Data Synthesis

We first tabulated the baseline characteristics of the included studies, documenting the reported outcomes and studies retained after accounting for overlap. For reviews with low overlap across two or more included systematic reviews, we extracted data from primary studies and performed random-effects meta-analysis to derive pooled effect estimates for relevant outcomes. For reviews with moderate-to-high overlap, data were extracted directly from the included systematic reviews (numbers of exposed, numbers of unexposed, and total sample size, or ORs or RRs). Results were presented in narrative form, as well as in forest plots and tables. For consistency, effect measures were converted to ORs where possible. For some outcomes, due to missing data, the pooled estimates could not be converted to ORs. R was used for the analysis.

## 3. Results

### 3.1. Literature Search

A literature search in Embase, PubMed, and the Cochrane Library identified 4026 potential reviews (Embase: 3027, PubMed: 765, and Cochrane: 54). We excluded 499 duplicate records. After screening 3527 titles and abstracts, 449 full texts were assessed, of which 385 were excluded, and the reasons for exclusion were recorded. Initially, 76 reviews were included to assess study quality and overlap.

### 3.2. Literature Screening

**Figure 1:**
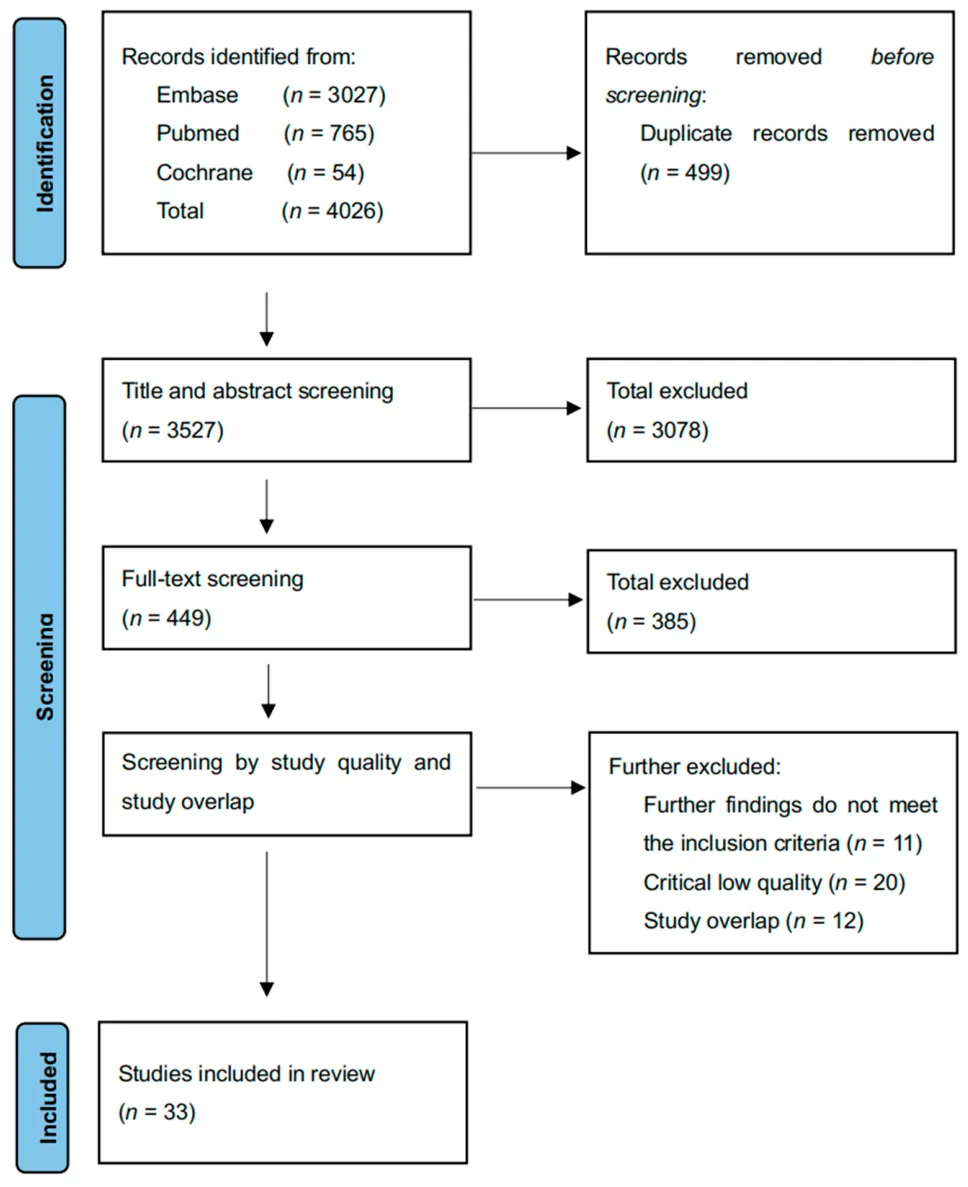
Selection process of relevant articles.

Of the initial 76 reviews included, 11 were excluded after reading the full text because they did not meet the inclusion criteria [8,9,10,11,12,13,14,15,16,17,18] and 20 were excluded due to critically low quality (assessed using the AMSTAR 2 tool) [19,20,21,22,23,24,25,26,27,28,29,30,31,32,33,34,35,36,37,38]. Twelve studies were excluded due to high overlap in CCA calculations [39,40,41,42,43,44,45,46,47,48,49,50]. Details on the excluded studies are provided in Supplementary Table S4. Ultimately, the study included 33 reviews. Figure 1 illustrates the selection process based on the PRISMA flowchart.

### 3.3. Findings of Quality Assessment

The quality assessment was conducted using the AMSTAR2 tool, with key areas including: protocol registered before the commencement of the review (Item 2), adequacy of the literature search (Item 4), justification for excluding individual studies (Item 7), risk of bias in individual studies included in the review (Item 9), appropriateness of meta-analytical methods (Item 11), consideration of risk of bias when interpreting the results of the review (Item 13), and assessment of the presence and potential impact of publication bias or small-study bias (Item 15). The 20 excluded reviews failed to meet the criteria in at least two critical domains. Only one of the included reviews obtained a high-quality rating according to the AMSTAR 2 checklist, while the rest were of moderate or low quality (Supplementary Table S2).

### 3.4. Overlapping and Non-Overlapping Associations

Most pregnancy outcomes reported for vitamin supplementation or level measurements were diverse. Among the 34 calculated overlapping associations, only one association had low overlap, while the rest were of high overlap, with the degree of overlap (CCA) ranging from 9.52% to 58.33%. Due to severe overlap, 12 meta-analyses were excluded. Details on the creation of the reference matrix and the calculation of CCA can be found in Supplementary Table S3.

### 3.5. Study Characteristics

All 33 included systematic reviews performed meta-analyses [3,4,5,6,51,52,53,54,55,56,57,58,59,60,61,62,63,64,65,66,67,68,69,70,71,72,73,74,75,76,77,78,79], with the total number of synthesized primary studies in each included meta-analysis ranging from 5 to 64. The characteristics of the included systematic reviews are presented in Table 1.

### 3.6. Summary of Results

#### 3.6.1. Levels

##### Vitamin B12

Vitamin B12 and GDM

Pregnant women with vitamin B12 deficiency had a higher risk of GDM in prospective cohort studies [OR 1.46, 95% CI (1.21, 1.79), *n* = 4; Table 2A], but no significant association was observed in cross-sectional and cohort studies [OR 0.97, 95% CI (0.76, 1.25), *n* = 6; Table 2A] [66]. There was no significant difference in serum vitamin B12 levels between pregnant women with GDM and healthy controls [67].

Vitamin B12 and Pre-eclampsia

Serum vitamin B12 levels were significantly lower in pregnant women with pre-eclampsia compared to healthy controls [WMD −15.24 pg/mL, 95% CI (−27.52, −2.95), *n* = 19; Table 2A] [68].

Vitamin B12 and Preterm Birth Rates

There was no significant difference in preterm birth rates between pregnant women with vitamin B12 deficiency and those with adequate levels [75].

Vitamin B12 and Neonatal Outcomes

Pregnant women with vitamin B12 deficiency had a higher risk of delivering low-birth-weight infants [RR 1.15, 95% CI (1.01, 1.31), *n* = 9; Table 2B], whereas there was no significant difference in the incidence of small-for-gestational-age infants [75].

##### Folic Acid

Compared with healthy controls, pregnant women with GDM had significantly lower serum folate levels [MD 0.73 ng/mL, 95% CI (0.23, 1.22), *n* = 17; Table 2A] and erythrocyte folate levels [MD 36.11 ng/mL, 95% CI (19.12, 53.09), *n* = 5; Table 2A] [59].

##### Vitamin C

Vitamin C and GDM

Pregnant women in the group with lower vitamin C levels had a significantly increased risk of GDM [OR 2.72, 95% CI (1.24, 4.19), *n* = 6; Table 2A], and vitamin C levels in women with GDM were significantly lower than those in the control group [SMD −0.71, 95% CI (−0.07, −0.36), *n* = 13; Table 2A] [58].

Vitamin C and Preterm Premature Rupture of Membranes (PPROM)

Patients with PPROM had significantly different serum vitamin C levels compared to the control group [Hedges’ g −1.48, 95% CI (−2.82, −0.14), *n* = 18; Table 2A] [55].

##### Vitamin D

Vitamin D is generally considered to be highly correlated with pregnancy outcomes, but the cutoff values defining vitamin D deficiency/insufficiency are not yet uniformly established across different studies. Most scholars define vitamin D deficiency as a blood vitamin D level < 20 ng/mL, insufficiency as a blood vitamin D level < 30 ng/mL, and sufficiency as a blood vitamin D level ≥ 30 ng/mL.

Vitamin D and Recurrent Spontaneous Abortion (RSA)

Compared with the “sufficient + insufficient” group, the incidence of RSA was more than three times higher in the “deficient” group [OR 4.02, 95% CI (2.23, 7.25), *n* = 12; Table 2A] [3]. Compared with the healthy control group, there was a significant difference in serum vitamin D levels among pregnant women with RSA [SMD −1.48, 95% CI (−2.01, −0.94), *n* = 14; Table 2A] [3].

Vitamin D and Miscarriage Rates

In comparison with the “sufficient” group, the “deficient + insufficient” group had a 60% higher miscarriage rate [OR 1.6, 95% CI (1.11, 2.30), *n* = 6; Table 2A] [61]. Furthermore, compared with the “sufficient” control group, the miscarriage rate in the “deficient” group was significantly higher by 94% [OR 1.94, 95% CI (1.25, 3.02), *n* = 4; Table 2A] [61].

Vitamin D and Anemia

Relative to the “sufficient + insufficient” group, the incidence of maternal anemia was 61% higher in the “deficiency” group [OR 1.61, 95% CI (1.41, 1.83), *n* = 8; Table 2A] [64].

Vitamin D and GDM

Compared with the “sufficient + insufficient” group, the prevalence of GDM was 53% higher in the “deficient” group [OR 1.53, 95% CI (1.33, 1.75), *n* = 20; Table 2A] [77]. Compared with the “sufficient” group, the prevalence of GDM was 55% higher in the vitamin D “deficient + insufficient” group [OR 1.55, 95% CI (1.21, 1.98), *n* = 8; Table 2A] [79]. Another study indicated that compared with the sufficient control group, the prevalence of GDM in the deficient group was 15% higher [OR 1.15, 95% CI (1.00, 1.30), *n* = 29; Table 2A] [74].

When comparing serum vitamin D levels with those of a healthy control group, pregnant women with GDM showed a significant difference [WMD −4.52 nmol/L, 95% CI (−5.19, −3.85), *n* = 21] [62,77] (Supplementary Figures S1 and S2) or [SMD −29.7, 95% CI (−36.6, −22.8), *n* = 18; Table 2A] [74]. Details of the reanalysis are presented in the Supplementary Materials.

Vitamin D and Gestational Hypertension or Pre-eclampsia

In comparison with the “sufficient + insufficient” group, the incidence of pre-eclampsia was 35% higher in the “deficient” group [OR 1.35, 95% CI (1.10, 1.66), *n* = 19; Table 2A] [5]. There was no significant difference in the prevalence of gestational hypertension [56]. Compared with the “sufficient” group, the “deficient + insufficient” group had a 58% higher incidence of pre-eclampsia [OR 1.58, 95% CI (1.39, 1.79), *n* = 14; Table 2A] [5].

Vitamin D and Preterm Birth Rates

Relative to the “insufficient + sufficient” group, the preterm birth rate was 28% higher in the vitamin D deficiency group [OR 1.28, 95% CI (1.08, 1.52), *n* = 21; Table 2A]. There was no significant difference in preterm birth rates between the vitamin D “deficient + insufficient” group and the “sufficient” group. When vitamin D deficiency was more strictly defined as a serum vitamin D level below 12 ng/mL, the preterm birth rate in the deficiency group increased by 23% [OR 1.23, 95% CI (1.05, 1.43), *n* = 9; Table 2A] [6].

Vitamin D and Prenatal Depression

Regarding the prevalence of prenatal depression, there was no significant difference in the rate of prenatal depression regardless of whether the vitamin D cutoff value was set at 12, 20, or 30 ng/mL [57,73]. However, compared with healthy controls, pregnant women with prenatal depression had significantly lower serum vitamin D levels [MD −4.63 ng/mL, 95% CI (−8.88, −0.38), *n* = 5; Table 2A] [57] or [SMD −0.41, 95% CI (−0.57, −0.25), *n* = 3; Table 2A] [53].

Vitamin D and Neonatal Outcomes

In the vitamin D deficiency group, the incidence of SGA infants increased by 43% compared with the “sufficient + insufficient” group [OR = 1.43, 95%CI (1.08, 1.91), *n* = 19; Table 2B] [6], while no significant differences were observed in neonatal birth length, birth weight and head circumference [6]. Other studies have shown that there were no significant differences in the incidence of SGA infants and birth weight between the vitamin D “deficient + insufficient” group and the “sufficient” group [6]. In addition, no significant difference in the incidence of SGA infants was found between the vitamin D deficiency group and the sufficient control group [54].

When vitamin D deficiency was defined as a serum vitamin D level less than 12 ng/mL, the incidence of SGA infants in the deficient group was significantly higher than that in the control group [OR 1.59, 95%CI (1.24–2.03), *n* = 11; Table 2B] [6]. Meanwhile, neonatal birth weight [MD −87.82 g, 95%CI (−119.73, −55.91), *n* = 15; Table 2B] and head circumference [MD −0.19 cm, 95%CI (−0.32, −0.06), *n* = 7; Table 2B] in the deficient group were also significantly lower than those in the control group, whereas no significant difference in neonatal birth length was noted between the two groups [6].

Vitamin D and Assisted Reproductive Outcomes

Compared with the “insufficient + sufficient” group, the clinical pregnancy rate in the deficient group decreased by 19% [OR 0.81, 95% CI (0.70, 0.95), *n* = 21; Table 2C], while, compared with the sufficient group, the clinical pregnancy rate in the “deficient + insufficient” group decreased by 29% [OR 0.71, 95% CI (0.55, 0.91), *n* = 16; Table 2C] [52].

Regardless of whether a cutoff value of 20 or 30 ng/mL was used, vitamin D showed no significant effect on the rates of ongoing pregnancy, live birth, biochemical pregnancy, miscarriage, or implantation in assisted reproductive technology outcomes [52,65]. Compared with the sufficient control group, the live birth rate [OR 0.57, 95% CI (0.43, 0.76), *n* = 6; Table 2C] and the ongoing pregnancy rate [OR 0.78, 95% CI (0.61, 0.98), *n* = 11; Table 2C] in the vitamin D deficiency group were significantly lower than those in the sufficient group [65]; however, there were no significant differences in clinical pregnancy rates [72] or miscarriage rates [65]. Furthermore, more refined comparative studies demonstrated that no significant differences were found in any assisted reproductive outcomes either between the deficiency group and the insufficient group, or between the insufficient group and the sufficient group [71].

##### Vitamin E

In comparison with the healthy control group, pregnant women with GDM showed a significant difference in serum vitamin E levels [MD −0.06 mg/dL, 95% CI (−0.06, −0.05), *n* = 11; Table 2A] [70].

#### 3.6.2. Supplementation

The summary of meta-analyses regarding the effects of vitamin supplementation in pregnant women on pregnancy outcomes is shown in Table 3. 

##### Vitamin A

Prenatal vitamin A supplementation has no effect on stillbirth rates, neonatal mortality, or maternal mortality [69].

##### Folic Acid

Folic acid supplementation reduces the incidence of megaloblastic anemia in pregnant women [RR 0.21, 95% CI (0.11, 0.38), *n* = 4] [51]; however, it has no significant effect on the incidence of low birth weight, stillbirth, preterm birth, gestational hypertension, or pre-eclampsia, and does not improve prenatal anemia [51,76].

##### Vitamin C

Vitamin C supplementation reduces the incidence of PPROM [RR 0.67, 95% CI (0.45, 0.99), *n* = 4] [55], but has no significant effect on the incidence of preterm birth or pre-eclampsia [78].

##### Vitamin D

Vitamin D supplementation reduces the incidence of GDM [RR 0.70, 95% CI (0.51, 0.95), *n* = 7] [4] and the rate of fetal death/stillbirth [RR 0.69, 95% CI (0.48, 0.99), *n* = 13] [63]. It also improves neonatal birth length [MD 0.27 cm, 95% CI (0.02, 0.51), *n* = 20] and birth weight [MD 37.07 g, 95% CI (9.67, 83.80), *n* = 33] [63]; however, it has no effect on the rates of low birth weight, macrosomia, preterm birth, or small-for-gestational-age infants, nor on birth head circumference [63]. Furthermore, vitamin D supplementation had no significant effect on various assisted reproductive outcomes, including the rate of continuing pregnancy [RR 0.86, 95% CI (0.71, 1.05), *n* = 3], the clinical pregnancy rate [RR 1.34, 95% CI (0.81, 2.24), *n* = 21], the miscarriage rate [RR 1.21, 95% CI (0.72, 2.04), *n* = 5], the rate of high-quality embryos [RR 1.09, 95% CI (0.82, 1.45), *n* = 5], the rate of biochemical pregnancy [RR 1.53, 95% CI (1.06, 2.20), *n* = 4], and the fertilization rate [RR 0.96, 95% CI (0.85, 1.09), *n* = 3] [60].

To date, no high-quality studies have been identified that examine the association between vitamin B12 and vitamin E supplementation and pregnancy outcomes.

## 4. Discussion

### 4.1. Principal Findings

The findings of this umbrella review indicate that vitamin deficiency or insufficiency during pregnancy is associated with various adverse maternal and fetal outcomes, including preterm birth, miscarriage, anemia, preterm rupture of membranes, GDM, pre-eclampsia, RSA, prenatal depression, birth head circumference, low birth weight and SGA, as well as ART outcomes including clinical pregnancy rates and live birth/pregnancy continuation rates.

Among maternal outcomes, RSA, miscarriage, and maternal anemia are primarily associated with vitamin D. Premature rupture of membranes is primarily associated with vitamin C. GDM is primarily associated with vitamin C and vitamin D. Although folic acid and vitamin E levels in patients with GDM are significantly lower than those in healthy control groups [59,70], there is no evidence indicating that supplementation with these two vitamins is effective for reducing the prevalence of GDM. Gestational hypertension and pre-eclampsia are primarily associated with vitamin D. Though vitamin B12 levels in patients with pre-eclampsia are significantly lower than those in healthy control groups [68], there is no evidence indicating that vitamin B12 supplementation is effective for reducing the prevalence of pre-eclampsia. Although women with prenatal depression exhibit significant differences in serum vitamin D levels [53], there is no evidence that the incidence of prenatal depression differs significantly between vitamin D-deficient or -insufficient groups and control groups [57], even when the cutoff value is strictly defined as 12 ng/mL [73]. Maternal mortality has no obvious relationship with vitamins.

Among neonatal outcomes, the incidence of low-birth-weight infants may be improved by vitamin B12 supplementation. Birth length, birth weight, birth head circumference, the incidence of stillbirth/fetal death, and the preterm birth rate can be improved by vitamin D supplementation. Although the incidence of SGA infants has a certain association with vitamin D levels [6], there is no evidence showing that vitamin D supplementation can improve it. Furthermore, no association was detected between the incidence of macrosomia and vitamins.

Most studies have only examined the association between vitamin D and assisted reproductive outcomes. Among these, only the vitamin D-deficient group showed a significant association with the ongoing pregnancy rate and live birth rate compared to the sufficient group [65]. Other outcomes such as the clinical pregnancy rate, miscarriage rate, high-quality embryo rate, biochemical pregnancy rate, implantation rate, and fertilization rate did not demonstrate clinical significance. In terms of the effects of supplementation interventions, the overall improvement in pregnancy outcomes from vitamin supplementation is limited. To facilitate visualization of the main outcomes, we have added a table summarizing the key statistical findings of this study (Table 4).

In observational and supplementation intervention studies, we identified two consistent and noteworthy associations. We found that when vitamin D deficiency was defined as serum 25(OH)D level < 12 ng/mL, newborn birth weight was significantly lower in the vitamin D-deficient group than in the control group [MD −87.82 g, 95%CI (−119.73, −55.91)] [6]. In addition, interventional studies demonstrated that vitamin D supplementation significantly increased neonatal birth weight [MD 37.07 g 95%CI (9.67, 83.80)] [63]. These findings suggest that vitamin D exerts a certain effect on neonatal birth weight. However, neonatal birth weight is determined by multiple factors. The clinical relevance of the birth weight gain achieved by vitamin D supplementation, as well as the clinical significance of fetal developmental impairment caused by vitamin D deficiency, remains to be confirmed by further studies.

Moreover, we observed that pregnant women with vitamin D insufficiency or deficiency had a significantly higher incidence of GDM. Appropriate vitamin D supplementation significantly reduced the risk of GDM [RR 0.70 95%CI (0.51, 0.95)] [4]. Given the high prevalence and complex pathogenesis of GDM, further large-scale, long-duration randomized controlled trials are required to verify the clinical effect of vitamin D on GDM. Only one meta-analysis was rated as having high systematic quality, while the rest were rated as moderate or low.

### 4.2. Comparison with the Existing Literature

#### 4.2.1. Vitamin D

The association between vitamin D and pregnancy outcomes is the strongest. Previous systematic reviews of observational studies have indicated that women with vitamin D deficiency are more susceptible to developing pre-eclampsia [42,80], although some studies have also suggested no significant association between vitamin D deficiency and pre-eclampsia [81,82]. Our analysis demonstrates that lower vitamin D levels correspond to a graded increase in the risk of pre-eclampsia. The underlying mechanism may be that elevated vitamin D levels improve trophoblast invasion, which is essential for normal placental formation [83]. Furthermore, an increasing body of evidence suggests that vitamin D has a positive impact on endothelial repair and angiogenesis [84,85], and that vitamin D deficiency is linked to the pathogenesis of cardiovascular diseases and arterial hypertension [86]. Therefore, it is highly likely that vitamin D plays a role in improving endothelial repair and angiogenesis, as well as in controlling blood pressure in pre-eclampsia.

The association between vitamin D and perinatal mood disorders has not yet been fully explored. Existing studies suggest that vitamin D may exert effects by participating in the synthesis of monoamine neurotransmitters [87,88], regulating intracellular and extracellular calcium homeostasis in neurons [89], and modulating the hypothalamic–pituitary–adrenal (HPA) axis [90].

The mechanisms by which vitamin D affects ART outcomes have not yet been fully determined. First, vitamin D may affect the development of follicles and embryos. In animal experiments, mice with the VDR gene knocked out exhibited impaired folliculogenesis and uterine hypoplasia [91]. Additionally, vitamin D may promote the production of steroid hormones such as estradiol, estrone, and progesterone [92], and it participates in the production of adult anti-Müllerian hormone (AMH), thereby regulating the recruitment of primary follicles [93,94]. Some studies have found that among infertile women, the concentration of vitamin D in the follicular fluid at the time of oocyte retrieval in the successful pregnancy group was significantly higher than that in the failed group, suggesting that higher vitamin D levels in the follicular fluid may be associated with better oocyte quality [95,96]. Second, vitamin D has been found to affect endometrial receptivity. At the animal experiment level, studies have shown that vitamin D receptors are expressed in the mouse endometrium, and female mice with vitamin D receptor mutations exhibit uterine hypoplasia and infertility [97,98]. At the molecular level, one study demonstrated that 1,25(OH)2D3 can upregulate the expression of HOXA10 mRNA and protein by binding to receptors in endometrial stromal cells, and HOXA10 is crucial for female fertility and embryo implantation [99]. Third, vitamin D has a potential antioxidant effect in human female gametes. Studies have shown that higher antioxidant concentrations are associated with improved ovarian stimulation efficiency and higher pregnancy success rates in infertile patients [100]. Furthermore, vitamin D can also affect implantation and early pregnancy through its immunomodulatory effects. Compared to vitamin D, evidence for the association between other vitamins and ART outcomes is relatively scarce. The research focus has been limited to clinical pregnancy rates, live birth rates, and ongoing pregnancy rates, lacking exploration of intermediate outcomes such as oocyte quality and embryo scoring. At the same time, sample sizes are generally small and study designs are highly heterogeneous, making it difficult for the existing evidence to form unified conclusions. Consequently, it cannot provide reliable support for individualized vitamin supplementation in the ART population.

Regarding supplementation dosage, there is currently no unified standard for vitamin D dosing in pregnancy. One randomized controlled trial demonstrated that daily vitamin D supplementation of 600 IU can correct vitamin D deficiency (defined as serum 25(OH)D < 12 ng/mL) among pregnant women [101]. The Institute of Medicine (IOM) recommended a daily vitamin D intake of 400–600 IU/day, with a tolerable upper intake level (UL) of 4000 IU/day [102]. Overall, prenatal vitamin D supplementation is relatively safe provided that serum 25(OH)D concentrations remain below 50 ng/mL.

#### 4.2.2. Vitamin B12 and Folic Acid

Vitamin B12 indirectly affects blood glucose by participating in the production, secretion and metabolism of insulin [103]. Our study indicates that the association between vitamin B12 and GDM varies depending on study design. Prospective cohort studies have observed a significantly increased risk of GDM in individuals with vitamin B12 deficiency, whereas cross-sectional studies and some cohort studies have not found this association. It is speculated that the core reason lies in differences in the timing of testing. Cross-sectional studies and some cohort studies often measure serum vitamin B12 levels during the mid-to-late stages of pregnancy, whereas in GDM patients, factors such as disease-related dietary intake at this stage may mask low vitamin B12 levels, thereby obscuring the true association between vitamin B12 and GDM.

Many studies have reported that folate and vitamin B12 may jointly participate in the progression of GDM [11,66]. Vitamin B12 and folate are essential for protein and DNA synthesis. Vitamin B12 deficiency or excessive folate impairs mitochondrial DNA synthesis, which may trigger insulin signaling dysfunction via mitochondrial stress and subsequently lead to insulin resistance [104]. Additionally, it can elevate homocysteine levels, further inducing insulin resistance and increasing the risk of GDM [105]. Therefore, the safe dosage range of folate during pregnancy requires further investigation. The US Preventive Services Task Force (USPSTF) recommends that, to prevent fetal neural tube defects, pregnant women should take a daily supplement of 400 to 800 micrograms of folic acid starting three months before conception and continue through at least the third month of pregnancy [106].

In our study, serum vitamin B12 levels were significantly lower in patients with pre-eclampsia compared with healthy controls. This suggests that monitoring serum vitamin B12 levels is valuable for early identification and clinical intervention of pre-eclampsia, and may serve as a potential biochemical marker for disease assessment. However, the exact underlying association remains unclear.

#### 4.2.3. Vitamin C

There is inconsistent evidence regarding the clinical value of vitamin C supplementation during pregnancy. Our study revealed that serum vitamin C levels were markedly lower in patients with premature rupture of membranes (PROM) and GDM than in the control group. Additionally, vitamin C levels were moderately negatively correlated with the prevalence of GDM. This may be attributed to the involvement of vitamin C in glucose transport in erythrocytes and its ability to alleviate oxidative stress, thereby improving insulin sensitivity [107,108].

Existing studies have demonstrated that PROM is associated with elevated oxidative stress as well as abnormal formation and structure of collagen [109]. Vitamin C plays a vital role in collagen metabolism, and collagen contributes to maintaining the mechanical strength and integrity of amniotic connective tissues [110,111]. While appropriate vitamin C supplementation has been proven to prevent PROM, high-dose vitamin C may induce excessive oxidative stress and further accelerate collagen degradation [112,113]. Accordingly, such potential adverse effects must be taken into account when determining the dosage of vitamin C supplementation. Furthermore, several studies have indicated that vitamin C supplementation may cause gastrointestinal discomfort [78].

#### 4.2.4. Vitamin E

As a fat-soluble antioxidant, there is far more evidence of the risks associated with vitamin E supplementation than of its benefits [21]. A Cochrane systematic review noted that vitamin E supplementation not only fails to prevent key adverse pregnancy outcomes but also increases the risk of abdominal pain and preterm rupture of membranes in pregnant women [114]. Its effects are uncertain only in terms of improving the incidence of GDM [70] and reducing the risk of placental abruption [115]. Our study found significant differences in vitamin E levels between pregnant women with GDM and healthy controls. The mechanism by which vitamin E deficiency promotes the onset and progression of GDM is similar to that of vitamin C and is associated with oxidative stress [70]. However, due to the potential risks associated with supplementation, its use is not routinely recommended in clinical practice.

#### 4.2.5. Vitamin A

The overall impact of vitamin A on pregnancy outcomes is not significant, but its “bidirectional risk” characteristic still warrants attention. Vitamin A deficiency increases the risk of fetal growth restriction, preterm birth, and night blindness in pregnant women [35], whereas excessive supplementation during early pregnancy may lead to fetal craniofacial, cardiac, and central nervous system malformations [116]. Therefore, routine additional vitamin A supplementation is not necessary during pregnancy and individualized intervention under medical supervision is required only in cases of confirmed deficiency. Strict dose control is central to clinical application of vitamin A.

### 4.3. Strengths and Limitations

The primary strengths of this review include the use of a comprehensive search strategy without time restrictions, periodic repetition of searches, and the inclusion of the most recent and highest-level evidence from meta-analyses. Additionally, we incorporated a broad range of vitamin levels or supplements, and adverse pregnancy outcomes. Through literature searches and expert consultation, this review was refined to improve its relevance and applicability.

The primary limitation of this umbrella review is that the effects of certain vitamin levels or supplementation on rare adverse pregnancy outcomes (e.g., HELLP syndrome, polyhydramnios or oligohydramnios) could not be included in the absence of relevant systematic reviews. Additionally, we did not examine the impact of vitamin supplementation or levels on long-term pregnancy outcomes.

Some meta-analyses were excluded due to a “very low” AMSTAR2 rating, resulting in missing outcomes such as neonatal respiratory distress, maternal infections, hyperemesis gravidarum, and fetal/neonatal congenital anomalies. Additionally, a limited number of studies (*n* = 3) were included in some meta-analyses, potentially introducing publication bias. Therefore, further primary research is needed to validate these findings. Only one of the included reviews achieved a high-quality rating according to the AMSTAR2 checklist, with the remainder rated as moderate or low quality, which impacts the overall certainty of evidence in this review.

Bias exists in the measurement and supplementation assessment of vitamins. For instance, gestational vitamin D status is commonly assessed by serum 25(OH)D concentrations. However, circulating vitamin D levels fluctuate daily according to diet, duration of sun exposure and sunlight intensity, and only reflect short-term vitamin D nutritional status. Therefore, although we excluded studies with combined micronutrient interventions (including iron, calcium, magnesium and other trace elements), factors such as ethnicity, socioeconomic status, residential latitude, seasonal sunlight variation, lifestyle and dietary patterns may affect baseline serum vitamin levels and confound the estimation of associations between vitamin supplementation and pregnancy or assisted reproductive outcomes. Furthermore, overlapping metabolic pathways among micronutrients may lead to unforeseen interactions and synergistic effects, which could influence the conclusions of this review.

Inconsistent cut-off values represent one of the major sources of bias in this work. For vitamin D, we strictly defined vitamin D deficiency and insufficiency based on current consensus. Nevertheless, there remains no universal agreement on the criteria for defining deficiency of other vitamins. Furthermore, although our findings suggest that supplementation with certain vitamins may improve maternal and infant outcomes, such benefits apply primarily to women with vitamin deficiency. For pregnant individuals with adequate baseline vitamin status, high-quality evidence supporting the benefits of supplementation remains lacking.

More importantly, although our findings are statistically significant, they cannot directly validate clinical applicability. For example, it remains unclear whether routine vitamin D supplementation should be recommended for patients with RSA, or routine vitamin C supplementation for women with GDM. Despite strong statistical associations reported in primary studies, the clinical utility of supplementation should be comprehensively judged by patient age, body mass index (BMI), baseline vitamin status, diet and lifestyle in clinical practice. A limitation of this work is the absence of formal certainty-of-evidence grading in the underlying systematic reviews, which precludes a comprehensive assessment of evidence certainty. Further in-depth mechanistic investigations and large-scale randomized controlled trials are warranted to clarify the effects of vitamins on maternal and infant health outcomes.

## 5. Conclusions

In summary, the association between different vitamins and pregnancy outcomes shows marked heterogeneity and is influenced by diagnostic criteria, study design, intervention timing and dosage. This review highlights the adverse outcomes associated with low levels of multiple vitamins in pregnancy and suggests that prenatal vitamin supplementation may reduce the risk of certain adverse events. In clinical practice, individualized supplementation regimens should be developed based on characteristics such as the pregnant woman’s baseline vitamin levels, stage of pregnancy, and whether the patient is receiving ART, to avoid indiscriminate supplementation. Further high-quality prospective studies and RCTs are warranted to clarify the mechanisms, dose–response relationships and target populations for each vitamin, supporting evidence-based clinical practice in prenatal vitamin management.

## Figures and Tables

**Table 1:** Characteristics of the included meta-analyses.

	First Author	Publication Year	Study Design	Types of Vitamins	No. of Studies in MA	Outcomes	AMSTAR-2
1	Xu [52]	2025	Observation	Vitamin D	23	Outcomes of assisted reproductive technology (ongoing pregnancy rate, live birth rate, clinical pregnancy rate, miscarriage rate, biochemical pregnancy rate and implantation rate)	Moderate
2	Yuan [53]	2024	Observation	Vitamin D	13	Perinatal depression	Low
3	You [54]	2024	Observation	Vitamin D	13	Neonatal outcomes (low-birth-weight infants, preterm birth and small for gestational age)	Low
4	Pejcic [55]	2024	Observation/RCT	Vitamin C	25	Premature/term preterm rupture of membranes	High
5	Cui [56]	2024	Observation/RCT	Vitamin D	17	Gestational hypertension	Low
6	Centeno [57]	2024	Observation	Vitamin D	25	Prenatal and postnatal depression or anxiety	Moderate
7	Zhou [58]	2023	Observation	Vitamin C	15	Gestational diabetes	Low
8	Xu [59]	2023	Observation	Folic acid	20	Gestational diabetes	Low
9	Zhou [60]	2022	RCT	Vitamin D	5	Outcomes of assisted reproductive technology (clinical pregnancy rate, miscarriage rate, ongoing pregnancy rate, biochemical pregnancy rate, fertilization rate and high-quality embryo rate)	Low
10	Tamblyn [61]	2022	Observation	Vitamin D	10	Miscarriage rate	Low
11	Moslehi [62]	2022	Observation	Vitamin D	7	Gestational diabetes	Low
12	Liu [63]	2022	RCT	Vitamin D	42	Neonatal outcomes (neonatal mortality rate, preterm birth rate, low-birth-weight infants, small for gestational age infants, macrosomic infants and congenital anomalies)	Low
13	Lima [64]	2022	Observation	Vitamin D	17	Gestational anemia	Moderate
14	Irwinda [4]	2022	RCT	Vitamin D	27	Pre-eclampsia, gestational diabetes, preterm birth and low birth weight	Low
15	Iliuta [65]	2022	Observation/RCT	Vitamin D	15	Outcomes of assisted reproductive technology (implantation rate, biochemical pregnancy rate, clinical pregnancy rate, miscarriage rate, ongoing pregnancy rate and live birth rate)	Low
16	Hu [5]	2022	Observation	Vitamin D	22	Pre-eclampsia	Moderate
17	He [66]	2022	Observation/RCT	Vitamin B12	10	Gestational diabetes	Low
18	Chen [67]	2023	Observation/RCT	Vitamin B12	15	Gestational diabetes	Low
19	Chen [3]	2022	Observation	Vitamin D	14	Recurrent miscarriage	Low
20	Mardali [68]	2021	Observation	Vitamin B12	19	Pre-eclampsia	Low
21	Keats [69]	2021	Observation	Vitamin D, Vitamin A	64	Maternal mortality rate, stillbirth rate, preterm birth, small for gestational age and cesarean section rate	Low
22	Tous [6]	2020	Observation	Vitamin D	54	Neonatal outcomes (birth weight, length, head circumference, small for gestational age and preterm birth)	Moderate
23	Sharifipour [70]	2020	Observation	Vitamin E	13	Gestational diabetes	Moderate
24	Cozzolino [71]	2020	Observation	Vitamin D	14	Outcomes of assisted reproductive technology (live birth rate/ongoing pregnancy rate, clinical pregnancy rate and miscarriage rate)	Moderate
25	Zhao [72]	2019	Observation	Vitamin D	9	Outcomes of assisted reproductive technology (live birth rate, ongoing pregnancy rate and clinical pregnancy rate)	Low
26	Wang [73]	2018	Observation	Vitamin D	9	Perinatal depression	Low
27	Nargesi [74]	2018	Observation	Vitamin D	29	Gestational diabetes	Low
28	Rogne [75]	2017	Observation	Vitamin B12	22	Neonatal outcomes (low birth weight, preterm birth and small for gestational age)	Moderate
29	Yang [76]	2016	Observation/RCT	Folic acid	11	Gestational hypertension and pre-eclampsia	Low
30	Zhang [77]	2015	Observation	Vitamin D	20	Gestational diabetes	Low
31	Rumbold [78]	2015	RCT	Vitamin C	29	Pre-eclampsia, preterm birth, preterm premature rupture of membranes, fetal death/stillbirth, maternal perinatal mortality, birth weight and intrauterine growth restriction	Low
32	Wei [79]	2013	Observation	Vitamin D	20	Pre-eclampsia, gestational diabetes, preterm birth and small for gestational age	Low
33	Lassi [51]	2013	Observation/RCT	Folic acid	31	Premature birth, stillbirth/neonatal death, low birth weight, improvement in antenatal anemia and megaloblastic anemia	Low

**Table 2:** Summary of meta-analyses on the effects of vitamin levels in pregnant women on pregnancy outcomes. (**A**) Maternal outcomes. (**B**) Offspring outcomes. (**C**) ART outcomes.

Types of Vitamins	Outcome	First Author	Publication Year	No. of Studies in MA	No. of Participants	Comparator (Vitamin Supplementation or Level)	Metric of MA	Effect Size	95% CI	I^2^	Methods of Assessing Publication Bias
(**A**)											
Vitamin B12	Gestational diabetes	Chen [67]	2023	12	6492	level	MD	−6.83	(−17.56, 3.89)	80.0%	Funnel plot, Egger’s test (*p* = 0.52)
		He [66] ^1^	2022	4	NA	deficiency	OR	1.46	(1.21, 1.79)	59.0%	NA
		He [66] ^2^	2022	6	NA	deficiency	OR	0.97	(0.76, 1.25)	81.6%	NA
	Pre-eclampsia	Mardali [68]	2021	19	3211	level	WMD	−15.24	(−27.52, −2.95)	97.8%	Funnel plot
Folic acid	Gestational diabetes	Xu [59] ^3^	2023	17	5983	level	MD	0.73	(0.23, 1.22)	47.0%	Funnel plot
		Xu [59] ^4^	2023	5	4314	level	MD	36.11	(19.12, 53.09)	96.0%	Funnel plot
Vitamin C	Premature rupture of membranes (preterm/term labor)	Pejcic [55]	2024	18	1220	level	Hedges’ g	−1.48	(−2.82, −0.14)	87.4%	Funnel plot, Egger’s test (*p* < 0.001), the Begg-Mazumdar test (*p* = 0.161)
	Gestational diabetes	Zhou [60]	2023	6	NA	level	OR	2.72	(1.24, 4.19)	58.3%	Funnel plot, Egger’s test
		Zhou [60]	2023	13	NA	level	SMD	−0.71	(−1.07, −0.36)	93.1%	Funnel plot, Egger’s test
Vitamin D	Gestational diabetes	- ^5^	-	21	6083	level	WMD	−4.52	(−5.19, −3.85)	57.3%	Funnel plot
		Nargesi [74]	2018	29	NA	deficiency vs. normal	OR	1.15	(1.00, 1.30)	31.6%	Funnel plot
		Nargesi [74]	2018	18	NA	level	SMD	−29.7	(−36.6, −22.8)	75.7%	Funnel plot
		Zhang [77]	2015	20	NA	<20 vs. ≥20 (ng/mL)	OR	1.53	(1.33, 1.75)	16.2%	Funnel plot, Begg’s test (*p* = 0.163), Egger’s test (*p* = 0.11)
		Wei [79]	2013	8	3840	<30 vs. ≥30 (ng/mL)	OR	1.55	(1.21, 1.98)	7.0%	Funnel plot
	Miscarriage	Tamblyn [61]	2022	6	6338	<30 vs. ≥30 (ng/mL)	OR	1.6	(1.11, 2.30)	35.0%	NA
		Tamblyn [61]	2022	4	3674	deficiency vs. normal	OR	1.94	(1.25, 3.02)	18.0%	NA
	Gestational hypertension	Cui [56]	2024	11	17,104	<20 vs. ≥20 (ng/mL)	RR	0.99	(0.97, 1.02)	67.5%	Egger’s test
		Cui [56]	2024	6	330,254	<20 vs. ≥20 (ng/mL)	RR	1.09	(0.84, 1.41)	75.4%	Egger’s test
	Pre-eclampsia	Hu [5]	2022	19	23,217	<20 vs. ≥20 (ng/mL)	OR	1.35	(1.10, 1.66)	57.0%	Funnel plot, Egger’s test (*p* = 0.069)
		Hu [5]	2022	14	17,719	<30 vs. ≥30 (ng/mL)	OR	1.58	(1.39, 1.79)	34.0%	Funnel plot, Egger’s test (*p* = 0.415)
	Gestational anemia	Lima [64]	2022	8	6530	<20 vs. ≥20 (ng/mL)	OR	1.61	(1.41, 1.83)	48.0%	NA
	Recurrent miscarriage	Chen [3]	2022	12	NA	<20 vs. ≥20 (ng/mL)	OR	4.02	(2.23, 7.25)	82.2%	Begg’s test (*p* = 0.012), Egger’s test (*p* = 0.026)
		Chen [3]	2022	14	3028	level	SMD	−1.48	(−2.01, −0.94)	97.3%	Begg’s test (*p* = 0.028), Egger’s test (*p* = 0.064)
	Prenatal depression	Centeno [57]	2024	5	5645	level	MD	−4.63	(−8.88, −0.38)	91.0%	NA
		Centeno [57]	2024	3	1424	<30 vs. ≥30 (ng/mL)	OR	1.51	(0.98, 2.30)	11.0%	NA
		Centeno [57]	2024	4	1577	<20 vs. ≥20 (ng/mL)	OR	2.16	(0.74, 6.37)	78.0%	NA
		Yuan [53]	2024	3	758	level	SMD	−0.41	(−0.57, −0.25)	30.0%	Begg’s test (*p* = 0.734), Egger’s test (*p* = 0.600)
		Wang [73]	2018	3	NA	<12 vs. ≥12 (ng/mL)	OR	1.47	(0.92, 2.36)	87.2%	Egger’s test
Vitamin E	Gestational diabetes	Sharifipour [70]	2020	11	596	level	MD	−0.06	(−0.06, −0.05)	94.0%	Funnel plot
(**B**)											
Vitamin B12	LBW infants	Rogne [75]	2017	9	9030	deficiency	RR	1.15	(1.01, 1.31)	5.0%	Funnel plot
	Preterm birth	Rogne [75]	2017	9	9102	deficiency	RR	1.21	(0.99, 1.49)	20.0%	Funnel plot
	SGA	Rogne [75]	2017	9	7934	deficiency	RR	1.05	(0.92, 1.19)	0.0%	Funnel plot
Vitamin D	Body length	Tous [6]	2020	4	3556	<12 vs. ≥12 (ng/mL)	MD	−0.28	(−0.66, 0.10)	69.0%	Funnel plot
		Tous [6]	2020	7	6929	<20 vs. ≥20 (ng/mL)	MD	−0.12	(−0.09, 0.33)	63.0%	Funnel plot
	Birth weight	Tous [6]	2020	15	16,042	<12 vs. ≥12 (ng/mL)	MD	−87.82	(−119.73, −55.91)	58.0%	Funnel plot
		Tous [6]	2020	13	15,379	<20 vs. ≥20 (ng/mL)	MD	−19.27	(−63.34, 24.80)	84.0%	Funnel plot
		Tous [6]	2020	5	6382	<30 vs. ≥30 (ng/mL)	MD	−15.15	(−12.73, 43.04)	27.0%	Funnel plot
	Birth HC	Tous [6]	2020	7	7114	<12 vs. ≥12 (ng/mL)	MD	−0.19	(−0.32, −0.06)	66.0%	Funnel plot
		Tous [6]	2020	7	5979	<20 vs. ≥20 (ng/mL)	MD	−0.47	(−1.11, 0.16)	98.0%	Funnel plot
	Preterm birth	Tous [6]	2020	21	26,937	<20 vs. ≥20 (ng/mL)	OR	1.28	(1.08, 1.52)	61.0%	Funnel plot
		Tous [6]	2020	10	18,034	<30 vs. ≥30 (ng/mL)	OR	1.18	(0.91, 1.54)	72.0%	Funnel plot
		Tous [6]	2020	11	19,505	<12 vs. ≥12 (ng/mL)	OR	1.23	(1.05, 1.43)	0%	Funnel plot
	SGA	You [54]	2024	9	27,240	deficiency vs. normal	OR	1.47	(0.81, 2.68)	89.0%	Funnel plot, Egger’s test
		Tous [6]	2020	19	28,414	<20 vs. ≥20 (ng/mL)	OR	1.43	(1.08, 1.91)	89.0%	Funnel plot
		Tous [6]	2020	7	9536	<30 vs. ≥30 (ng/mL)	OR	0.98	(0.81, 1.17)	39.0%	Funnel plot
		Tous [6]	2020	11	17,486	<12 vs. ≥12 (ng/mL)	OR	1.59	(1.24, 2.03)	71.0%	Funnel plot
(**C**)											
Vitamin D	Ongoing pregnancy rate	Xu [52]	2025	5	4443	<20 vs. ≥20 (ng/mL)	OR	0.99	(0.87, 1.12)	0.0%	Funnel plot, Egger’s test (*p* = 0.035)
		Xu [52]	2025	5	4918	<30 vs. ≥30 (ng/mL)	OR	0.96	(0.79, 1.17)	0.0%	Funnel plot, Egger’s test (*p* = 0.367)
		Iliuta [65]	2022	11	2132	deficiency vs. normal	OR	0.78	(0.61, 0.98)	25.7%	NA
	Live birth rate	Xu [52]	2025	7	2345	<20 vs. ≥20 (ng/mL)	OR	0.84	(0.62, 1.13)	54.0%	Funnel plot, Egger’s test (*p* = 0.035)
		Xu [52]	2025	6	1947	<30 vs. ≥30 (ng/mL)	OR	0.69	(0.54, 0.89)	15.0%	Funnel plot, Egger’s test (*p* = 0.367)
		Iliuta [65]	2022	6	1013	deficiency vs. normal	OR	0.57	(0.43, 0.76)	0.0%	NA
	Ongoing pregnancy rate/live birth rate	Cozzolino [71]	2020	5	812	insufficiency vs. normal	OR	0.80	(0.59, 1.08)	0.0%	NA
		Cozzolino [71]	2020	5	821	deficiency vs. insufficiency	OR	1.07	(0.81, 1.42)	0.0%	NA
	Clinical pregnancy rate	Xu [52]	2025	21	11,642	<20 vs. ≥20 (ng/mL)	OR	0.81	(0.70, 0.95)	62.0%	Funnel plot, Egger’s test (*p* = 0.035)
		Xu [52]	2025	16	7450	<30 vs. ≥30 (ng/mL)	OR	0.71	(0.55, 0.91)	54.0%	Funnel plot, Egger’s test (*p* = 0.367)
		Cozzolino [71]	2020	5	812	insufficiency vs. normal	OR	0.77	(0.48, 1.21)	51.0%	NA
		Cozzolino [71]	2020	5	887	deficiency vs. insufficiency	OR	0.93	(0.69, 1.24)	0.0%	NA
		Zhao [72]	2019	9	2254	deficiency vs. normal	RR	0.91	(0.77, 1.07)	68.0%	Funnel plot
	Miscarriage rate	Xu [52]	2025	5	2747	<20 vs. ≥20 (ng/mL)	OR	1.05	(0.83, 1.32)	38.0%	Funnel plot, Egger’s test (*p* = 0.035)
		Iliuta [65]	2022	9	NA	<30 vs. ≥30 (ng/mL)	OR	1.09	(0.83, 1.44)	0.0%	NA
		Iliuta [65]	2022	11	2042	deficiency vs. normal	OR	1.00	(0.72, 1.38)	12.6%	NA
	Biochemical pregnancy rate	Xu [52]	2025	3	826	<20 vs. ≥20 (ng/mL)	OR	0.94	(0.45, 1.96)	64.0%	Funnel plot, Egger’s test (*p* = 0.035)
		Xu [52]	2025	3	826	<30 vs. ≥30 (ng/mL)	OR	0.68	(0.33, 1.39)	55.0%	Funnel plot, Egger’s test (*p* = 0.367)
	Implantation rate	Xu [52]	2025	5	5710	<30 vs. ≥30 (ng/mL)	OR	0.8	(0.62, 1.03)	47.0%	Funnel plot, Egger’s test (*p* = 0.367)

LBW: low birth weight; HC: head circumference; SGA: small for gestational age. ^1^ Prospective cohort study, ^2^ cross-sectional study and cohort study, ^3^ serum folate levels, ^4^ erythrocyte folate levels, ^5^ this row lists the content that was reanalyzed due to low overlap. As a result of missing partial raw data and statistical limitations, the pooled estimates cannot be fully converted to ORs.

**Table 3:** Summary of meta-analyses on the effects of vitamin supplementation in pregnant women on pregnancy outcomes.

Types of Vitamins	Outcome	First Author	Publication Year	No. of Studies in MA	No. of Participants	Comparator (Vitamin Supplementation or Level)	Metric of MA	Effect Size	95% CI	I^2^	Methods of Assessing Publication Bias
Vitamin A	Neonatal outcome (stillbirth)	Keats [69]	2021	3	115,223	Supplementation	RR	1.01	(0.96, 1.07)	0.0%	NA
	Maternal mortality rate	Keats [69]	2021	3	124,002	Supplementation	RR	0.90	(0.68, 1.18)	35.0%	NA
Folic acid	Gestational hypertension	Yang [76]	2016	5	NA	Supplementation	RR	1.03	(0.98, 1.09)	80.4%	Egger’s test
	Neonatal outcome (LBW infants)	Lassi [51]	2013	4	3113	Supplementation	RR	0.83	(0.66, 1.04)	22.0%	Funnel plot
	Neonatal outcome (stillbirth)	Lassi [51]	2013	3	3110	Supplementation	RR	1.33	(0.96, 1.85)	0.0%	Funnel plot
	Neonatal outcome (preterm birth)	Lassi [51]	2013	3	2959	Supplementation	RR	1.01	(0.73, 1.38)	2.0%	Funnel plot
	Pre-eclampsia	Yang [76]	2016	8	NA	Supplementation	RR	0.99	(0.90, 1.08)	74.5%	Egger’s test
	Improvement in antenatal anemia	Lassi [51]	2013	8	4149	Supplementation	RR	0.62	(0.35, 1.10)	90.0%	Funnel plot
	Megaloblastic anemia	Lassi [51]	2013	4	3839	Supplementation	RR	0.21	(0.11, 0.38)	15.0%	Funnel plot
Vitamin C	Premature rupture of membranes in preterm labor	Pejcic [55]	2024	4	457	Supplementation	RR	0.67	(0.45, 0.99)	0.0%	Funnel plot, Egger’s test (*p* < 0.001), the Begg-Mazumdar test (*p* = 0.337)
	Neonatal outcome (preterm birth)	Rumbold [78]	2015	5	1685	Supplementation	RR	1.06	(0.75, 1.48)	47.1%	Funnel plot
	Pre-eclampsia	Rumbold [78]	2015	3	1191	Supplementation	RR	0.88	(0.48, 1.61)	0.0%	Funnel plot
Vitamin D	Outcomes of ART (ongoing pregnancy rate)	Zhou [60]	2022	3	747	Supplementation	RR	0.86	(0.71, 1.05)	0.0%	NA
	Outcomes of ART (clinical pregnancy rate)	Zhou [60]	2022	4	838	Supplementation	RR	1.34	(0.81, 2.24)	68.0%	NA
	Outcomes of ART (miscarriage rate)	Zhou [60]	2022	3	303	Supplementation	RR	1.21	(0.72, 2.04)	0.0%	NA
	Outcomes of ART (quality embryo rate)	Zhou [60]	2022	5	938	Supplementation	RR	1.09	(0.82, 1.45)	0.0%	NA
	Outcomes of ART (biochemical pregnancy rate)	Zhou [60]	2022	4	303	Supplementation	RR	1.53	(1.06, 2.20)	2.0%	NA
	Outcomes of ART (fertilization rate)	Zhou [60]	2022	3	806	Supplementation	RR	0.96	(0.85, 1.09)	9.0%	NA
	Gestational diabetes	Irwinda [4]	2022	7	1722	Supplementation	RR	0.70	(0.51, 0.95)	0.0%	Funnel plot
	Neonatal outcome (LBW infants)	Liu [63]	2022	10	3925	Supplementation	RR	0.90	(0.66, 1.24)	31.8%	Funnel plot
	Neonatal outcome (body length)	Liu [63]	2022	20	6668	Supplementation	MD	0.27	(0.02, 0.51)	63.1%	Funnel plot
	Neonatal outcome (stillbirth)	Liu [63]	2022	13	6238	Supplementation	RR	0.69	(0.48, 0.99)	0.0%	Funnel plot
	Neonatal outcome (birth weight)	Liu [63]	2022	33	9322	Supplementation	MD	37.07	(9.67, 83.80)	77.1%	Funnel plot
	Neonatal outcome (birth HC)	Liu [63]	2022	18	6318	Supplementation	MD	0.15	(−0.02, 0.32)	68.3%	Funnel plot
	Neonatal outcome (macrosomic infant)	Liu [63]	2022	3	894	Supplementation	RR	1.10	(0.60, 2.01)	0.0%	Funnel plot
	Neonatal outcome (preterm birth)	Liu [63]	2022	21	8488	Supplementation	RR	0.94	(0.79, −1.09)	8.7%	Funnel plot
	Neonatal outcome (SGA)	Liu [63]	2022	10	3245	Supplementation	RR	0.84	(0.63, 1.10)	20.4%	Funnel plot

ART: Assisted reproductive technology; LBW: low birth weight; HC: head circumference; SGA: small for gestational age.

**Table 4:** Summary of meta-analyses with statistically significant associations.

Types of Vitamins	Outcome	Comparator (Vitamin Supplementation or Level)	Metric of MA	Effect Size	95% CI
Vitamin D	Recurrent miscarriage	<20 vs. ≥20 (ng/mL)	OR	4.02	(2.23, 7.25)
Vitamin C	Gestational diabetes	level	OR	2.72	(1.24, 4.19)
Vitamin D	Miscarriage	deficiency vs. normal	OR	1.94	(1.25, 3.02)
Vitamin D	Live birth rate	deficiency vs. normal	OR	0.57	(0.43, 0.76)
Vitamin D	Gestational anemia	<20 vs. ≥20 (ng/mL)	OR	1.61	(1.41, 1.83)
Vitamin D	Miscarriage	<30 vs. ≥30 (ng/mL)	OR	1.6	(1.11, 2.30)
Vitamin D	SGA	<12 vs. ≥12 (ng/mL)	OR	1.59	(1.24, 2.03)
Vitamin D	Pre-eclampsia	<30 vs. ≥30 (ng/mL)	OR	1.58	(1.39, 1.79)
Vitamin D	Gestational diabetes	<30 vs. ≥30 (ng/mL)	OR	1.55	(1.21, 1.98)
Vitamin D	Gestational diabetes	<20 vs. ≥20 (ng/mL)	OR	1.53	(1.33, 1.75)
Vitamin B12	Gestational diabetes	deficiency	OR	1.46	(1.21, 1.79)
Vitamin D	Live birth rate	<30 vs. ≥30 (ng/mL)	OR	0.69	(0.54, 0.89)
Vitamin D	SGA	<20 vs. ≥20 (ng/mL)	OR	1.43	(1.08, 1.91)
Vitamin D	Clinical pregnancy rate	<30 vs. ≥30 (ng/mL)	OR	0.71	(0.55, 0.91)
Vitamin D	Pre-eclampsia	<20 vs. ≥20 (ng/mL)	OR	1.35	(1.10, 1.66)
Vitamin D	Ongoing pregnancy rate	deficiency vs. normal	OR	0.78	(0.61, 0.98)
Vitamin D	Preterm birth	<20 vs. ≥20 (ng/mL)	OR	1.28	(1.08, 1.52)
Vitamin D	Preterm birth	<12 vs. ≥12 (ng/mL)	OR	1.23	(1.05, 1.43)
Vitamin D	Clinical pregnancy rate	<20 vs. ≥20 (ng/mL)	OR	0.81	(0.70, 0.95)
Vitamin D	Gestational diabetes	deficiency vs. normal	OR	1.15	(1.00, 1.30)
Folic acid	Megaloblastic anemia	Supplementation	RR	0.21	(0.11, 0.38)
Vitamin C	Premature rupture of membranes in preterm labor	Supplementation	RR	0.67	(0.45, 0.99)
Vitamin D	Neonatal outcome (stillbirth)	Supplementation	RR	0.69	(0.48, 0.99)
Vitamin D	Gestational diabetes	Supplementation	RR	0.7	(0.51, 0.95)
Vitamin B12	LBW infants	deficiency	RR	1.15	(1.01, 1.31)
Vitamin D	Neonatal outcome (birth weight)	Supplementation	MD	37.07	(9.67, 83.80)
Vitamin D	Neonatal outcome (body length)	Supplementation	MD	0.27	(0.02, 0.51)
Vitamin D	Neonatal outcome (birth HC)	<12 vs. ≥12 (ng/mL)	MD	−0.19	(−0.32, −0.06)
Vitamin D	Neonatal outcome (birth weight)	<12 vs. ≥12 (ng/mL)	MD	−87.82	(−119.73, −55.91)

SGA: small for gestational age; LBW: low birth weight; HC: head circumference.

## Data Availability

The data described in the manuscript, along with the codebook and analytic code, are available from the corresponding author upon reasonable request.

## Supplementary Materials

The following supporting information can be downloaded at https://www.mdpi.com/article/10.3390/jcm15218434/s1, Figure S1: Forest plots of the patients with GDM vs healthy controls; Figure S2: Funnel plot to access the publication bias; Table S1: Search Strategy; Table S2: Quality Assessment of included studies using AMSTAR 2 Tool; Table S3: Overlapping and non-overlapping associations by corrected covered area (CCA) calculations; Table S4: List of excluded studies; Table S5: Prisma 2020 checklist; Table S6: Prisma 2020 for abstract checklist.

## References

[1] Crider K.S., Qi Y.P., Yeung L.F., Mai C.T., Head Zauche L., Wang A., Daniels K., Williams J.L. (2022). Folic Acid and the Prevention of Birth Defects: 30 Years of Opportunity and Controversies. Annu. Rev. Nutr..

[2] De-Regil L.M., Peña-Rosas J.P., Fernández-Gaxiola A.C., Rayco-Solon P. (2015). Effects and Safety of Periconceptional Oral Folate Supplementation for Preventing Birth Defects. https://pubmed.ncbi.nlm.nih.gov/26662928/.

[3] Chen C., Wang S., Zhang C., Wu X., Zhou L., Zou X., Guan T., Zhang Z., Hao J. (2022). Association between serum vitamin D level during pregnancy and recurrent spontaneous abortion: A systematic review and meta-analysis. Am. J. Reprod. Immunol..

[4] Irwinda R., Hiksas R., Lokeswara A.W., Wibowo N. (2022). Vitamin D supplementation higher than 2000 IU/day compared to lower dose on maternal-fetal outcome: Systematic review and meta-analysis. Women’s Health.

[5] Hu K.-L., Zhang C.-X., Chen P., Zhang D., Hunt S. (2022). Vitamin D Levels in Early and Middle Pregnancy and Preeclampsia, a Systematic Review and Meta-Analysis. Nutrients.

[6] Tous M., Villalobos M., Iglesias L., Fernández-Barrés S., Arija V. (2020). Vitamin D status during pregnancy and offspring outcomes: A systematic review and meta-analysis of observational studies. Eur. J. Clin. Nutr..

[7] Huang Y., Chen Z., Chen B., Li J., Yuan X., Li J., Wang W., Dai T., Chen H., Wang Y. (2023). Dietary sugar consumption and health: Umbrella review. BMJ.

[8] Moghib K., Ghanm T.I., Abunamoos A., Rajabi M., Moawad S.M., Mohsen A., Kasem S., Elsayed K., Sayed M., Dawoud A.I. (2024). Efficacy of vitamin D supplementation on the incidence of preeclampsia: A systematic review and meta-analysis. BMC Pregnancy Childbirth.

[9] Amini S., Sheykhon A.A., Abiri B., Jafarirad S. (2024). Maternal Dietary Intakes and Risk of Spontaneous Abortion: A Systematic Review. Curr. Nutr. Food Sci..

[10] Walker K.C., Thorsteinsdottir F., Christesen H.T., Hjortdal V.E., Heitmann B.L., Specht I.O., Händel M.N. (2023). Vitamin D Supplementation and Vitamin D Status during Pregnancy and the Risk of Congenital Anomalies—A Systematic Review and Meta-Analysis. Nutrients.

[11] Li N., Jiang J., Guo L. (2022). Effects of maternal folate and vitamin B12 on gestational diabetes mellitus: A dose-response meta-analysis of observational studies. Eur. J. Clin. Nutr..

[12] Wang L., Hou Y., Meng D., Yang L., Meng X., Liu F. (2021). Vitamin B12 and Folate Levels During Pregnancy and Risk of Gestational Diabetes Mellitus: A Systematic Review and Meta-Analysis. Front. Nutr..

[13] Zhao R., Zhou L., Wang S., Xiong G., Hao L. (2022). Association between maternal vitamin D levels and risk of adverse pregnancy outcomes: A systematic review and dose-response meta-analysis. Food Funct..

[14] Zhao R., Zhou L., Wang S., Yin H., Yang X., Hao L. (2022). Effect of maternal vitamin D status on risk of adverse birth outcomes: A systematic review and dose–response meta-analysis of observational studies. Eur. J. Nutr..

[15] Rouhani P., Mokhtari E., Lotfi K., Saneei P. (2023). The association between circulating 25-hydroxyvitamin D levels and preeclampsia: A systematic review and dose-response meta-analysis of epidemiologic studies with GRADE assessment. Nutr. Rev..

[16] Tesfay N., Hailu G., Habtetsion M., Woldeyohannes F. (2023). Birth prevalence and risk factors of neural tube defects in Ethiopia: A systematic review and meta-analysis. BMJ Open.

[17] Wakoya R., Afework M. (2023). Burden of Neural Tube Defects and Their Associated Factors in Africa: A Systematic Review and Meta-Analysis. Int. J. Pediatr..

[18] Yu M., Shen Q. (2023). Effects of maternal folic acid supplementation on renal urinary system development in human offspring—A meta-analysis and systemic review. Pediatr. Med..

[19] Poel Y.H.M., Hummel P., Lips P., Stam F., van der Ploeg T., Simsek S. (2012). Vitamin D and gestational diabetes: A systematic review and meta-analysis. Eur. J. Intern. Med..

[20] Boskabadi H., Zakerihamidi M., Mehrad-Majd H., Ghoflchi S. (2025). Evaluation of vitamin D in the diagnosis of infants with respiratory distress, the clinical value: A systematic review and meta-analysis. Paediatr. Respir. Rev..

[21] Wang S., Shi M., Zhou L., Huang H., Mo S. (2023). Correlation of vitamin E level during pregnancy with maternal and neonatal health outcomes: A meta-analysis and systematic review. Am. J. Transl. Res..

[22] Fatima K., Asif M., Nihal K., Hussain H.U., Hasan A.W., Zahid M., Burney M.H., Asad F., Fatima S., Saleem M.B. (2022). Association between vitamin D levels in early pregnancy and gestational diabetes mellitus: A systematic review and meta-analysis. J. Fam. Med. Prim. Care.

[23] Milajerdi A., Abbasi F., Mousavi S.M., Esmaillzadeh A. (2021). Maternal vitamin D status and risk of gestational diabetes mellitus: A systematic review and meta-analysis of prospective cohort studies. Clin. Nutr..

[24] Chan K.Y., Wong M.M.H., Pang S.S.H., Lo K.K.H. (2021). Dietary supplementation for gestational diabetes prevention and management: A meta-analysis of randomized controlled trials. Arch. Gynecol. Obstet..

[25] Wang L., Zhang C., Song Y., Zhang Z. (2020). Serum vitamin D deficiency and risk of gestational diabetes mellitus: A meta-analysis. Arch. Med. Sci..

[26] Oh C., Keats E.C., Bhutta Z.A. (2020). Vitamin and Mineral Supplementation During Pregnancy on Maternal, Birth, Child Health and Development Outcomes in Low- and Middle-Income Countries: A Systematic Review and Meta-Analysis. Nutrients.

[27] Lassi Z.S., Kedzior S.G., Tariq W., Jadoon Y., Das J.K., Bhutta Z.A. (2020). Effects of Preconception Care and Periconception Interventions on Maternal Nutritional Status and Birth Outcomes in Low- and Middle-Income Countries: A Systematic Review. Nutrients.

[28] Tripathi P., Rao Y., Pandey K., Gautam K.A. (2019). Significance of Vitamin D on the susceptibility of gestational diabetes mellitus—A meta-analysis. Indian J. Endocrinol. Metab..

[29] Shen C., Wang L., Wu X., Mao S., Fang C. (2019). The relationship between Vitamin D and IVF: A systematic review and meta-analysis. Clin. Exp. Obstet. Gynecol..

[30] Hu L., Zhang Y., Wang X., You L., Xu P., Cui X., Zhu L., Ji C., Guo X., Wen J. (2018). Maternal Vitamin D Status and Risk of Gestational Diabetes: A Meta-Analysis. Cell. Physiol. Biochem..

[31] Amraei M., Mohamadpour S., Sayehmiri K., Mousavi S.F., Shirzadpour E., Moayeri A. (2018). Effects of Vitamin D Deficiency on Incidence Risk of Gestational Diabetes Mellitus: A Systematic Review and Meta-analysis. Front. Endocrinol..

[32] Zhang H., Huang Z., Xiao L., Jiang X., Chen D., Wei Y. (2017). Meta-analysis of the effect of the maternal vitamin D level on the risk of spontaneous pregnancy loss. Int. J. Gynecol. Obstet..

[33] Qin L.-L., Lu F.-G., Yang S.-H., Xu H.-L., Luo B.-A. (2016). Does maternal Vitamin D deficiency increase the risk of preterm birth: A meta-analysis of observational studies. Nutrients.

[34] Lu M., Xu Y., Lv L., Zhang M. (2016). Association between vitamin D status and the risk of gestational diabetes mellitus: A meta-analysis. Arch. Gynecol. Obstet..

[35] McCauley M.E., van den Broek N., Dou L., Othman M. (2015). Vitamin A supplementation during pregnancy for maternal and newborn outcomes. Cochrane Database Syst. Rev..

[36] Hyppönen E., Cavadino A., Williams D., Fraser A., Vereczkey A., Fraser W.D., Bánhidy F., Lawlor D., Czeizel A.E. (2013). Vitamin D and pre-eclampsia: Original data, systematic review and meta-analysis. Ann. Nutr. Metab..

[37] Aghajafari F., Nagulesapillai T., Ronksley P.E., Tough S.C., O’Beirne M., Rabi D.M. (2013). Association between maternal serum 25-hydroxyvitamin D level and pregnancy and neonatal outcomes: Systematic review and meta-analysis of observational studies. BMJ.

[38] Thorne-Lyman A., Fawzi W.W. (2012). Vitamin D during pregnancy and maternal, neonatal and infant health outcomes: A systematic review and meta-analysis. Paediatr. Perinat. Epidemiol..

[39] Quotah O.F., Andreeva D., Nowak K.G., Dalrymple K.V., Almubarak A., Patel A., Vyas N., Cakir G.S., Heslehurst N., Bell Z. (2024). Interventions in preconception and pregnant women at risk of gestational diabetes; a systematic review and meta-analysis of randomised controlled trials. Diabetol. Metab. Syndr..

[40] Palacios C., Kostiuk L.L., Cuthbert A., Weeks J. (2024). Vitamin D supplementation for women during pregnancy. Cochrane Database Syst. Rev..

[41] Lian R.-H., Qi P.-A., Yuan T., Yan P.-J., Qiu W.-W., Wei Y., Hu Y.-G., Yang K.-H., Yi B. (2021). Systematic review and meta-Analysis of vitamin D deficiency in different pregnancy on preterm birth: Deficiency in middle pregnancy might be at risk. Medicine.

[42] Aguilar-Cordero M.J., Lasserrot-Cuadrado A., Mur-Villar N., León-Ríos X.A., Rivero-Blanco T., Pérez-Castillo I.M. (2020). Vitamin D, preeclampsia and prematurity: A systematic review and meta-analysis of observational and interventional studies. Midwifery.

[43] Maugeri A., Barchitta M., Blanco I., Agodi A. (2019). Effects of Vitamin D supplementation during pregnancy on birth size: A systematic review and meta-analysis of randomized controlled trials. Nutrients.

[44] Chu J., Gallos I., Tobias A., Tan B., Eapen A., Coomarasamy A. (2018). Vitamin D and assisted reproductive treatment outcome: A systematic review and meta-analysis. Hum. Reprod..

[45] Bi W.G., Nuyt A.M., Weiler H., Leduc L., Santamaria C., Wei S.Q. (2018). Association Between Vitamin D Supplementation During Pregnancy and Offspring Growth, Morbidity, and Mortality: A Systematic Review and Meta-analysis. JAMA Pediatr..

[46] Zhou S.-S., Tao Y.-H., Huang K., Zhu B.-B., Tao F.-B. (2017). Vitamin D and risk of preterm birth: Up-to-date meta-analysis of randomized controlled trials and observational studies. J. Obstet. Gynaecol. Res..

[47] Chen Y., Zhu B., Wu X., Li S., Tao F. (2017). Association between maternal Vitamin D deficiency and small for gestational age: Evidence from a meta-analysis of prospective cohort studies. BMJ Open.

[48] Lv S.S., Wang J.Y., Wang X.Q., Wang Y., Xu Y. (2016). Serum vitamin D status and in vitro fertilization outcomes: A systematic review and meta-analysis. Arch. Gynecol. Obstet..

[49] De-Regil L.M., Palacios C., Lombardo L.K., Peña-Rosas J.P. (2016). Vitamin D supplementation for women during pregnancy. Cochrane Database Syst. Rev..

[50] De-Regil L.M., Palacios C., Ansary A., Kulier R., Peña-Rosas J.P. (2012). Vitamin D supplementation for women during pregnancy. Cochrane Database Syst. Rev..

[51] Lassi Z.S., Salam R.A., Haider B.A., Bhutta Z.A. (2013). Folic acid supplementation during pregnancy for maternal health and pregnancy outcomes. Cochrane Database Syst. Rev..

[52] Xu C., An X., Tang X., Yang Y., Deng Q., Kong Q., Hu Y., Yuan D. (2025). Association Between Vitamin D Level and Clinical Outcomes of Assisted Reproductive Treatment: A Systematic Review and Dose-Response Meta-Analysis. Reprod. Sci..

[53] Yuan Y., Qu L., Sun Q., He P., Zhou X. (2024). The Association between Vitamin D Deficiency and Perinatal Depression: A Systematic Review and Meta-Analysis. Alpha Psychiatry.

[54] You Z., Mei H., Zhang Y., Song D., Zhang Y., Liu C. (2024). The effect of vitamin D deficiency during pregnancy on adverse birth outcomes in neonates: A systematic review and meta-analysis. Front. Pediatr..

[55] Pejcic A.V., Petrovic N.Z., Djordjic M.D., Milosavljevic M.N. (2024). Vitamin C Levels in Pregnant Women and the Efficacy of Vitamin C Supplements in Preventing Premature Rupture of Membranes: A Systematic Review and Meta-Analysis. Balk. Med. J..

[56] Cui C., Huang X., Cui Y., Jiang S., Yao X., Li X. (2024). Vitamin D May not be Crucial: Association between Serum Vitamin D Level and Pregnancy-Induced Hypertension-A Meta-Analysis. Clin. Exp. Obstet. Gynecol..

[57] Centeno L.O.L., Fernandez M.D.S., Muniz F.W.M.G., Longoni A., de Assis A.M. (2024). Is Serum Vitamin D Associated with Depression or Anxiety in Ante- and Postnatal Adult Women? A Systematic Review with Meta-Analysis. Nutrients.

[58] Zhou L., Liu J., Zhou M. (2023). A comprehensive meta-analysis on the association between vitamin C intake and gestational diabetes mellitus: Insights and novel perspectives. Medicine.

[59] Xu R., Liu S., Zhong Z., Guo Y., Xia T., Chen Y., Ding L. (2023). The Influence of Maternal Folate Status on Gestational Diabetes Mellitus: A Systematic Review and Meta-Analysis. Nutrients.

[60] Zhou X., Wu X., Luo X., Shao J., Guo D., Deng B., Wu Z. (2022). Effect of Vitamin D Supplementation on In Vitro Fertilization Outcomes: A Trial Sequential Meta-Analysis of 5 Randomized Controlled Trials. Front. Endocrinol..

[61] Tamblyn J.A., Pilarski N.S.P., Markland A.D., Marson E.J., Devall A., Hewison M., Morris R.K., Coomarasamy A. (2022). Vitamin D and miscarriage: A systematic review and meta-analysis. Fertil. Steril..

[62] Moslehi N., Sakak F.R., Teymoori F., Tehrani F.R., Mirmiran P., Azizi F. (2022). The role of nutrition in the development and management of gestational diabetes among Iranian women: A systematic review and meta-analysis. J. Diabetes Metab. Disord..

[63] Liu Y., Ding C., Xu R., Wang K., Zhang D., Pang W., Tu W., Chen Y. (2022). Effects of vitamin D supplementation during pregnancy on offspring health at birth: A meta-analysis of randomized controlled trails. Clin. Nutr..

[64] Lima M.S., Pereira M., Castro C.T., Santos D.B. (2022). Vitamin D deficiency and anemia in pregnant women: A systematic review and meta-analysis. Nutr. Rev..

[65] Iliuta F., Pijoan J.I., Lainz L., Exposito A., Matorras R. (2022). Women’s vitamin D levels and IVF results: A systematic review of the literature and meta-analysis, considering three categories of vitamin status (replete, insufficient and deficient). Hum. Fertil..

[66] He J., Jiang D., Cui X., Ji C. (2022). Vitamin B12 status and folic acid/vitamin B12 related to the risk of gestational diabetes mellitus in pregnancy: A systematic review and meta-analysis of observational studies. BMC Pregnancy Childbirth.

[67] Chen X., Du Y., Xia S., Li Z., Liu J. (2023). Vitamin B(12) and gestational diabetes mellitus: A systematic review and meta-analysis. Br. J. Nutr..

[68] Mardali F., Fatahi S., Alinaghizadeh M., Kord Varkaneh H., Sohouli M.H., Shidfar F., Găman M.-A. (2021). Association between abnormal maternal serum levels of vitamin B12 and preeclampsia: A systematic review and meta-analysis. Nutr. Rev..

[69] Keats E.C., Oh C., Chau T., Khalifa D.S., Imdad A., Bhutta Z.A. (2021). Effects of vitamin and mineral supplementation during pregnancy on maternal, birth, child health and development outcomes in low- and middle-income countries: A systematic review. Campbell Syst. Rev..

[70] Sharifipour F., Abedi P., Ciahkal S.F., Jahanfar S., Mohaghegh Z., Zahedian M. (2020). Serum vitamin E level and gestational diabetes mellitus: A systematic review and meta-analysis. J. Diabetes Metab. Disord..

[71] Cozzolino M., Busnelli A., Pellegrini L., Riviello E., Vitagliano A. (2020). How vitamin D level influences in vitro fertilization outcomes: Results of a systematic review and meta-analysis. Fertil. Steril..

[72] Zhao J., Huang X., Xu B., Yan Y., Zhang Q., Li Y. (2018). Whether vitamin D was associated with clinical outcome after IVF/ICSI: A systematic review and meta-analysis. Reprod. Biol. Endocrinol..

[73] Wang J., Liu N., Sun W., Chen D., Zhao J., Zhang W. (2018). Association between vitamin D deficiency and antepartum and postpartum depression: A systematic review and meta-analysis of longitudinal studies. Arch. Gynecol. Obstet..

[74] Nargesi S., Ghorbani A., Shirzadpour E., Mohamadpour M., Mousavi S.F., Amraei M. (2018). A systematic review and meta-analysis of the association between Vitamin D deficiency and gestational diabetes mellitus. Biomed. Res. Ther..

[75] Rogne T., Tielemans M.J., Chong M.F.-F., Yajnik C.S., Krishnaveni G.V., Poston L., Jaddoe V.W.V., Steegers E.A.P., Joshi S., Chong Y.-S. (2017). Associations of Maternal Vitamin B12 Concentration in Pregnancy with the Risks of Preterm Birth and Low Birth Weight: A Systematic Review and Meta-Analysis of Individual Participant Data. Am. J. Epidemiol..

[76] Yang X., Chen H., Du Y., Wang S., Wang Z. (2016). Periconceptional folic acid fortification for the risk of gestational hypertension and pre-eclampsia: A meta-analysis of prospective studies. Matern. Child Nutr..

[77] Zhang M.-X., Pan G.-T., Guo J.-F., Li B.-Y., Qin L.-Q., Zhang Z.-L. (2015). Vitamin D Deficiency Increases the Risk of Gestational Diabetes Mellitus: A Meta-Analysis of Observational Studies. Nutrients.

[78] Rumbold A., Ota E., Nagata C., Shahrook S., Crowther C.A. (2015). Vitamin C supplementation in pregnancy. Cochrane Database Syst. Rev..

[79] Wei S.-Q., Qi H.-P., Luo Z.-C., Fraser W.D. (2013). Maternal vitamin D status and adverse pregnancy outcomes: A systematic review and meta-analysis. J. Matern Fetal Neonatal Med...

[80] Tabesh M., Salehi-Abargouei A., Tabesh M., Esmaillzadeh A. (2013). Maternal vitamin D status and risk of pre-eclampsia: A systematic review and meta-analysis. J. Clin. Endocrinol. Metab..

[81] Liu Y.-H., Zhang Y.-S., Chen J.-Y., Wang Z.-J., Liu Y.-X., Li J.-Q., Xu X.-J., Xie N.-J., Lye S., Tan N. (2023). Comparative effectiveness of prophylactic strategies for preeclampsia: A network meta-analysis of randomized controlled trials. Am. J. Obstet. Gynecol..

[82] Shand A.W., Nassar N., Von Dadelszen P., Innis S.M., Green T.J. (2010). Maternal vitamin D status in pregnancy and adverse pregnancy outcomes in a group at high risk for pre-eclampsia. BJOG Int. J. Obstet. Gynaecol..

[83] Chan S.Y., Susarla R., Canovas D., Vasilopoulou E., Ohizua O., McCabe C.J., Hewison M., Kilby M.D. (2015). Vitamin D promotes human extravillous trophoblast invasion in vitro. Placenta.

[84] Brodowski L., Burlakov J., Myerski A.C., von Kaisenberg C.S., Grundmann M., Hubel C.A., von Versen-Höynck F. (2014). Vitamin D prevents endothelial progenitor cell dysfunction induced by sera from women with preeclampsia or conditioned media from hypoxic placenta. PLoS ONE.

[85] Schröder-Heurich B., von Hardenberg S., Brodowski L., Kipke B., Meyer N., Borns K., von Kaisenberg C.S., Brinkmann H., Claus P., von Versen-Höynck F. (2019). Vitamin D improves endothelial barrier integrity and counteracts inflammatory effects on endothelial progenitor cells. FASEB J..

[86] Pilz S., Tomaschitz A., Ritz E., Pieber T.R. (2009). Vitamin D status and arterial hypertension: A systematic review. Nat. Rev. Cardiol..

[87] Newmark H.L., Newmark J. (2007). Vitamin D and Parkinson’s disease—A hypothesis. Mov. Disord..

[88] Pertile R.A.N., Cui X., Eyles D.W. (2016). Vitamin D signaling and the differentiation of developing dopamine systems. Neuroscience.

[89] Harms L.R., Cowin G., Eyles D.W., Kurniawan N.D., McGrath J.J., Burne T.H.J. (2012). Neuroanatomy and psychomimetic-induced locomotion in C57BL/6J and 129/X1SvJ mice exposed to developmental vitamin D deficiency. Behav. Brain. Res..

[90] Obradovic D., Gronemeyer H., Lutz B., Rein T. (2006). Cross-talk of vitamin D and glucocorticoids in hippocampal cells. J. Neurochem..

[91] Yoshizawa T., Handa Y., Uematsu Y., Takeda S., Sekine K., Yoshihara Y., Kawakami T., Arioka K., Sato H., Uchiyama Y. (1997). Mice lacking the vitamin D receptor exhibit impaired bone formation, uterine hypoplasia and growth retardation after weaning. Nat. Genet..

[92] Parikh G., Varadinova M., Suwandhi P., Araki T., Rosenwaks Z., Poretsky L., Seto-Young D. (2010). Vitamin D regulates steroidogenesis and insulin-like growth factor binding protein-1 (IGFBP-1) production in human ovarian cells. Horm. Metab. Res..

[93] Dennis N.A., Houghton L.A., Jones G.T., van Rij A.M., Morgan K., McLennan I.S. (2012). The level of serum anti-Müllerian hormone correlates with vitamin D status in men and women but not in boys. J. Clin. Endocrinol. Metab..

[94] Irani M., Merhi Z. (2014). Role of vitamin D in ovarian physiology and its implication in reproduction: A systematic review. Fertil. Steril..

[95] Polyzos N.P., Anckaert E., Guzman L., Schiettecatte J., Van Landuyt L., Camus M., Smitz J., Tournaye H. (2014). Vitamin D deficiency and pregnancy rates in women undergoing single embryo, blastocyst stage, transfer (SET) for IVF/ICSI. Hum. Reprod..

[96] Farzadi L., Khayatzadeh Bidgoli H., Ghojazadeh M., Bahrami Z., Fattahi A., Latifi Z., Shahnazi V., Nouri M. (2015). Correlation between follicular fluid 25-OH vitamin D and assisted reproductive outcomes. Iran. J. Reprod. Med..

[97] Johnson L.E., DeLuca H.F. (2001). Vitamin D receptor null mutant mice fed high levels of calcium are fertile. J. Nutr..

[98] Zarnani A.H., Shahbazi M., Salek-Moghaddam A., Zareie M., Tavakoli M., Ghasemi J., Rezania S., Moravej A., Torkabadi E., Rabbani H. (2010). Vitamin D3 receptor is expressed in the endometrium of cycling mice throughout the estrous cycle. Fertil. Steril..

[99] Lerchbaum E., Obermayer-Pietsch B. (2012). Vitamin D and fertility: A systematic review. Eur. J. Endocrinol..

[100] Velthut A., Zilmer M., Zilmer K., Kaart T., Karro H., Salumets A. (2013). Elevated blood plasma antioxidant status is favourable for achieving IVF/ICSI pregnancy. Reprod. Biomed. Online.

[101] Roth D.E., Morris S.K., Zlotkin S., Gernand A.D., Ahmed T., Shanta S.S., Papp E., Korsiak J., Shi J., Islam M.M. (2018). Vitamin D Supplementation in Pregnancy and Lactation and Infant Growth. N. Engl. J. Med..

[102] Ross A.C., Taylor C.L., Yaktine A.L., Del Valle H.B., Institute of Medicine (US) Committee to Review Dietary Reference Intakes for Vitamin D and Calcium (2011). Dietary Reference Intakes for Calcium and Vitamin D.

[103] Adaikalakoteswari A., Vatish M., Alam M.T., Ott S., Kumar S., Saravanan P. (2017). Low Vitamin B12 in Pregnancy Is Associated With Adipose-Derived Circulating miRs Targeting PPARγ and Insulin Resistance. J. Clin. Endocrinol. Metab..

[104] Zheng L.D., Linarelli L.E., Liu L., Wall S.S., Greenawald M.H., Seidel R.W., Estabrooks P.A., Almeida F.A., Cheng Z. (2015). Insulin resistance is associated with epigenetic and genetic regulation of mitochondrial DNA in obese humans. Clin. Epigenet..

[105] Cho N.H., Lim S., Jang H.C., Park H.K., Metzger B.E. (2005). Elevated homocysteine as a risk factor for the development of diabetes in women with a previous history of gestational diabetes mellitus: A 4-year prospective study. Diabetes Care.

[106] Barry M.J., Nicholson W.K., Silverstein M., Chelmow D., Coker T.R., Davis E.M., Donahue K.E., Jaén C.R., Li L., US Preventive Services Task Force (2023). Folic Acid Supplementation to Prevent Neural Tube Defects: US Preventive Services Task Force Reaffirmation Recommendation Statement. JAMA.

[107] Garcia-Diaz D.F., Campion J., Milagro F.I., Paternain L., Solomon A., Martinez J.A. (2009). Ascorbic acid oral treatment modifies lipolytic response and behavioural activity but not glucocorticoid metabolism in cafeteria diet-fed rats. Acta Physiol..

[108] Tu H., Li H., Wang Y., Niyyati M., Wang Y., Leshin J., Levine M. (2015). Low Red Blood Cell Vitamin C Concentrations Induce Red Blood Cell Fragility: A Link to Diabetes Via Glucose, Glucose Transporters, and Dehydroascorbic Acid. EBioMedicine.

[109] Casanueva E., Ripoll C., Tolentino M., Morales R.M., Pfeffer F., Vilchis P., Vadillo-Ortega F. (2005). Vitamin C supplementation to prevent premature rupture of the chorioamniotic membranes: A randomized trial. Am. J. Clin. Nutr..

[110] Tejero E., Perichart O., Pfeffer F., Casanueva E., Vadillo-Ortega F. (2003). Collagen synthesis during pregnancy, vitamin C availability, and risk of premature rupture of fetal membranes. Int. J. Gynecol. Obstet..

[111] Bryant-Greenwood G.D. (1998). The extracellular matrix of the human fetal membranes: Structure and function. Placenta.

[112] Maymon E., Romero R., Pacora P., Gervasi M.T., Bianco K., Ghezzi F., Yoon B.H. (2000). Evidence for the participation of interstitial collagenase (matrix metalloproteinase 1) in preterm premature rupture of membranes. Am. J. Obstet. Gynecol..

[113] Woods J.R. (2001). Reactive oxygen species and preterm premature rupture of membranes—A review. Placenta.

[114] Rumbold A., Duley L., Crowther C., Haslam R. (2008). Antioxidants for preventing pre-eclampsia. Cochrane Database Syst. Rev..

[115] Rumbold A., Ota E., Hori H., Miyazaki C., Crowther C.A. (2015). Vitamin E supplementation in pregnancy. Cochrane Database Syst. Rev..

[116] Bastos Maia S., Rolland Souza A.S., Costa Caminha M.d.F., Lins da Silva S., Callou Cruz R.d.S.B.L., Carvalho dos Santos C., Batista Filho M. (2019). Vitamin A and Pregnancy: A Narrative Review. Nutrients.

