# Supplementary material for: The Effect of Vitamins During Pregnancy on Pregnancy and Assisted Reproduction Outcomes: An Umbrella Review
Source: J Clin Med. 2026 Oct 29;15(21):8434. doi: 10.3390/jcm15218434 (PMC13763765; doi:10.3390/jcm15218434)

Table S1 Search Strategy

**Pubmed**	
Search number	Query
1	vitamin[Title/Abstract]
2	(((((((Pteroylglutamic Acid[Title/Abstract]) OR (Vitamin M[Title/Abstract])) OR (Vitamin B9[Title/Abstract])) OR (B9, Vitamin[Title/Abstract])) OR (Folacin[Title/Abstract])) OR (Folvite[Title/Abstract])) OR (Folic Acid[Title/Abstract])) OR (Folate[Title/Abstract])
3	#1 OR #2
4	((Complication, Pregnancy[Title/Abstract]) OR (Pregnancy Complication[Title/Abstract])) OR (Complications, Pregnancy[Title/Abstract])
5	((((((((((Eclampsia[Title/Abstract]) OR (Pre Eclampsia[Title/Abstract])) OR (Preeclampsia[Title/Abstract])) OR (Pre-eclampsia[Title/Abstract])) OR (Hypertension, Pregnancy Induced[Title/Abstract])) OR (Pregnancy-Induced Hypertension[Title/Abstract])) OR (Gestational Hypertension[Title/Abstract])) OR (Hypertension, Gestational[Title/Abstract])) OR (Pregnancy Induced Hypertension[Title/Abstract])) OR (Transient Hypertension, Pregnancy[Title/Abstract])) OR (Pregnancy Transient Hypertension[Title/Abstract])
6	(((((((Diabetes Mellitus, Gestational[Title/Abstract]) OR (Gestational Diabetes Mellitus[Title/Abstract])) OR (Diabetes, Pregnancy-Induced[Title/Abstract])) OR (Diabetes, Pregnancy Induced[Title/Abstract])) OR (Pregnancy-Induced Diabetes[Title/Abstract])) OR (Gestational Diabetes*[Title/Abstract])) OR (Resistance, Insulin[Title/Abstract])) OR (Insulin resistance[Title/Abstract])
7	((((Emesis[Title/Abstract]) OR (vomiting[Title/Abstract])) OR (Hyperemesis Gravidarum[Title/Abstract])) OR (Pernicious Vomiting of Pregnancy[Title/Abstract])) OR (Pregnancy Pernicious Vomiting[Title/Abstract])
8	((((((Infection, Reproductive Tract[Title/Abstract]) OR (Infections, Reproductive Tract[Title/Abstract])) OR (Reproductive Tract Infection[Title/Abstract])) OR (Genital Tract Infections[Title/Abstract])) OR (Genital Tract Infection[Title/Abstract])) OR (Infection, Genital Tract[Title/Abstract])) OR (Infections, Genital Tract[Title/Abstract])
10	anemia[Title/Abstract]

11	((((((((Premature rupture of membranes[Title/Abstract]) OR (Premature Rupture of Fetal Membranes[Title/Abstract])) OR (Premature Rupture of Membrane (Pregnancy[Title/Abstract]))) OR (Membrane Premature Rupture (Pregnancy[Title/Abstract]))) OR (Membrane Premature Ruptures (Pregnancy[Title/Abstract]))) OR (PROM (Pregnancy[Title/Abstract]))) OR (Preterm Premature Rupture of Fetal Membranes[Title/Abstract])) OR (Preterm PROM (Pregnancy[Title/Abstract]))) OR (PROM, Preterm (Pregnancy[Title/Abstract]))
12	(((Placental abruption[Title/Abstract]) OR (Abruption, Placental[Title/Abstract])) OR (Abruptions, Placental[Title/Abstract])) OR (Placental Abruptions[Title/Abstract])
13	((Disease*, Thyroid[Title/Abstract]) OR (Thyroid[Title/Abstract])) OR (Thyroid Disease*[Title/Abstract])
14	(((Cesarean Section*[Title/Abstract]) OR (Abdominal Delivery*[Title/Abstract])) OR (Delivery*, Abdominal[Title/Abstract])) OR (Postcesarean Section[Title/Abstract])
15	(Prolongation of labour[Title/Abstract]) OR (Prolonged labour[Title/Abstract])
16	(((((((postpartum haemorrhage[Title/Abstract]) OR (Hemorrhage, Postpartum[Title/Abstract])) OR (Delayed Postpartum Hemorrhage[Title/Abstract])) OR (Hemorrhage, Delayed Postpartum[Title/Abstract])) OR (Postpartum Hemorrhage, Delayed[Title/Abstract])) OR (Immediate Postpartum Hemorrhage[Title/Abstract])) OR (Hemorrhage, Immediate Postpartum[Title/Abstract])) OR (Postpartum Hemorrhage, Immediate[Title/Abstract])
17	(((((((Abortions, Spontaneous[Title/Abstract]) OR (Spontaneous Abortion*[Title/Abstract])) OR (Abortion*, Tubal[Title/Abstract])) OR (Tubal Abortion*[Title/Abstract])) OR (Miscarriage*[Title/Abstract])) OR (Early Pregnancy Loss*[Title/Abstract])) OR (Loss*, Early Pregnancy[Title/Abstract])) OR (Pregnancy Loss*, Early[Title/Abstract])
18	(((((Labor, Premature Obstetric[Title/Abstract]) OR (Labor, Premature[Title/Abstract])) OR (Premature Labor[Title/Abstract])) OR (Premature Obstetric Labor[Title/Abstract])) OR (Preterm Labor[Title/Abstract])) OR (Labor, Preterm[Title/Abstract])
19	((((Death*, Fetal[Title/Abstract]) OR (Fetal Death*[Title/Abstract])) OR (Fetal Demise[Title/Abstract])) OR (Demise, Fetal[Title/Abstract])) OR (stillbirth[Title/Abstract])
20	(((Average gestational age at delivery[Title/Abstract]) OR (Average gestational age at birth[Title/Abstract])) OR (Mean gestational age at delivery[Title/Abstract])) OR (Average foetal age at delivery[Title/Abstract])

21	Average birth weight[Title/Abstract]
22	(((Apgar score[Title/Abstract]) OR (Respiratory Distress Syndrome, Infant[Title/Abstract])) OR (Infantile Respiratory Distress Syndrome[Title/Abstract])) OR (Neonatal Respiratory Distress Syndrome[Title/Abstract])
23	(((((((((Abnormality*, Congenital[Title/Abstract]) OR (Congenital Abnormality*[Title/Abstract])) OR (Birth Defect*[Title/Abstract])) OR (Defect*, Birth[Title/Abstract])) OR (Congenital Defect*[Title/Abstract])) OR (Defect*, Congenital[Title/Abstract])) OR (Deformity*[Title/Abstract])) OR (Fetal Anomalies[Title/Abstract])) OR (Anomaly, Fetal[Title/Abstract])) OR (Fetal Malformation*[Title/Abstract])) OR (Malformation, Fetal[Title/Abstract])
24	((((((((((((((((Postpartum Depression[Title/Abstract]) OR (Post-Natal Depression[Title/Abstract])) OR (Depression, Post-Natal[Title/Abstract])) OR (Post Natal Depression[Title/Abstract])) OR (Post-Partum Depression[Title/Abstract])) OR (Depression, Post-Partum[Title/Abstract])) OR (Post Partum Depression[Title/Abstract])) OR (Postnatal Depression[Title/Abstract])) OR (Depression, Postnatal[Title/Abstract])) OR (Postnatal Dysphoria[Title/Abstract])) OR (Dysphoria, Postnatal[Title/Abstract])) OR (Postpartum Dysphoria[Title/Abstract])) OR (Dysphoria, Postpartum[Title/Abstract])) OR (Post-Partum Dysphoria[Title/Abstract])) OR (Dysphoria, Post-Partum[Title/Abstract])) OR (Post Partum Dysphoria[Title/Abstract])) OR (Post-Natal Dysphoria[Title/Abstract])) OR (Dysphoria, Post-Natal[Title/Abstract])) OR (Post Natal Dysphoria[Title/Abstract])
25	(((Death*, Perinatal[Title/Abstract]) OR (Perinatal Deaths[Title/Abstract])) OR (Neonatal Death*[Title/Abstract])) OR (Death*, Neonatal[Title/Abstract])
26	(((((((((Reproductive Technique[Title/Abstract]) OR (Technique*, Reproductive[Title/Abstract])) OR (Reproduction Technic*[Title/Abstract])) OR (Technic*, Reproduction[Title/Abstract])) OR (Reproduction Technique*[Title/Abstract])) OR (Technique*, Reproduction[Title/Abstract])) OR (Reproductive Technology[Title/Abstract])) OR (Technologies, Reproductive[Title/Abstract])) OR (Reproductive Technologies[Title/Abstract])) OR (Technology, Reproductive[Title/Abstract])
27	((((Biochemical pregnancy[Title/Abstract]) OR (Clinical pregnancy rate*[Title/Abstract])) OR (Clinical gestation rate*[Title/Abstract])) OR (Clinical live birth rate*[Title/Abstract])) OR (Clinical live-birth rate*[Title/Abstract])
28	(((((meta-analysis[Publication Type]) OR (meta analysis*[Publication Type])) OR (systematic review[Publication Type])) OR (systematic review*[Title/Abstract])) OR (systematic evolution*[Title/Abstract])) OR (meta analysis as topic[MeSH Terms])

29	#4 OR #5 OR #6 OR #7 OR #8 OR #10 OR #11 OR #12 OR #13 OR #14 OR #15 OR #16 OR #17 OR #18 OR #19 OR #20 OR #21 OR #22 OR #23 OR #24 OR #25 OR #26 OR #27
30	#3 AND #29 AND #28
**Embase**	
No.	Query Results
#1.	'vitamin'/exp
#2.	'folic acid'/exp OR 'pteroylglutamic acid':ti,ab,kw OR 'vitamin m':ti,ab,kw OR 'vitamin b9':ti,ab,kw OR 'b9, vitamin':ti,ab,kw OR 'folacin':ti,ab,kw OR 'folvite':ti,ab,kw OR 'folate':ti,ab,kw
#3.	'pregnancy complications'/exp OR 'pregnancy complication':ti,ab,kw OR 'complications, pregnancy':ti,ab,kw OR 'adverse birth outcomes':ti,ab,kw OR 'adverse birth outcome':ti,ab,kw OR 'birth outcome, adverse':ti,ab,kw OR 'outcome, adverse birth':ti,ab,kw
#4.	'eclampsia'/exp
#5.	'pre-eclampsia'/exp OR 'pre eclampsia':ti,ab,kw OR 'preeclampsia':ti,ab,kw
#6.	'hypertension, pregnancy-induced'/exp OR 'pregnancy-induced hypertension':ti,ab,kw OR 'gestational hypertension':ti,ab,kw OR 'hypertension, gestational':ti,ab,kw OR 'pregnancy induced hypertension':ti,ab,kw OR 'hypertensions, pregnancy induced':ti,ab,kw OR 'induced hypertension, pregnancy':ti,ab,kw OR 'induced hypertensions, pregnancy':ti,ab,kw OR 'transient hypertension, pregnancy':ti,ab,kw OR 'hypertension, pregnancy transient':ti,ab,kw OR 'pregnancy transient hypertension':ti,ab,kw
#7.	'diabetes, gestational'/exp OR 'diabetes mellitus, gestational':ti,ab,kw OR 'gestational diabetes mellitus':ti,ab,kw OR 'diabetes, pregnancy-induced':ti,ab,kw OR 'diabetes, pregnancy induced':ti,ab,kw OR 'pregnancy-induced diabetes':ti,ab,kw OR 'gestational diabetes':ti,ab,kw
#8.	'insulin resistance'/exp OR 'resistance, insulin':ti,ab,kw
#9.	'vomiting'/exp OR 'emesis':ti,ab,kw
#10.	'hyperemesis gravidarum'/exp OR 'pernicious vomiting of pregnancy':ti,ab,kw OR 'pregnancy pernicious vomiting':ti,ab,kw
#11.	#9 OR #10
#12.	'reproductive tract infections'/exp OR 'infection, reproductive tract':ti,ab,kw OR 'infections, reproductive tract':ti,ab,kw OR 'reproductive tract infection':ti,ab,kw OR 'genital tract infections':ti,ab,kw OR 'genital tract infection':ti,ab,kw OR 'infection, genital tract':ti,ab,kw OR 'infections, genital tract':ti,ab,kw
#13.	'anemia'/exp OR 'anemias':ti,ab,kw
#14.	'fetal membranes, premature rupture'/exp OR 'premature rupture of membranes':ti,ab,kw OR 'premature rupture of fetal membranes':ti,ab,kw OR 'premature rupture of membrane (pregnancy)':ti,ab,kw OR 'membrane premature rupture (pregnancy)':ti,ab,kw OR 'membrane premature ruptures

	(pregnancy)':ti,ab,kw OR 'prom (pregnancy)':ti,ab,kw OR 'preterm premature rupture of fetal membranes':ti,ab,kw OR 'preterm prom (pregnancy)':ti,ab,kw OR 'prom, preterm (pregnancy)':ti,ab,kw
#15.	'abruptio placentae'/exp OR 'placental abruption':ti,ab,kw OR 'abruption, placental':ti,ab,kw OR 'abruptions, placental':ti,ab,kw OR 'placental abruptions':ti,ab,kw
#16.	'thyroid diseases'/exp OR 'diseases, thyroid':ti,ab,kw OR 'disease, thyroid':ti,ab,kw OR 'thyroid disease':ti,ab,kw
#17.	'cesarean section'/exp OR 'cesarean sections':ti,ab,kw OR 'abdominal delivery':ti,ab,kw OR 'c-section (ob)':ti,ab,kw OR 'c section (ob)':ti,ab,kw OR 'c-sections (ob)':ti,ab,kw OR 'caesarean section':ti,ab,kw OR 'caesarean sections':ti,ab,kw OR 'delivery, abdominal':ti,ab,kw OR 'abdominal deliveries':ti,ab,kw OR 'deliveries, abdominal':ti,ab,kw OR 'postcesarean section':ti,ab,kw
#18.	'prolongation of labour':ti,ab,kw OR 'prolonged labour':ti,ab,kw
#19.	'postpartum hemorrhage'/exp OR 'hemorrhage, postpartum':ti,ab,kw OR 'delayed postpartum hemorrhage':ti,ab,kw OR 'hemorrhage, delayed postpartum':ti,ab,kw OR 'postpartum hemorrhage, delayed':ti,ab,kw OR 'immediate postpartum hemorrhage':ti,ab,kw OR 'hemorrhage, immediate postpartum':ti,ab,kw OR 'postpartum hemorrhage, immediate':ti,ab,kw
#20.	'abortion, spontaneous'/exp OR 'spontaneous abortions':ti,ab,kw OR 'spontaneous abortion':ti,ab,kw OR 'abortion, tubal':ti,ab,kw OR 'abortions, tubal':ti,ab,kw OR 'tubal abortion':ti,ab,kw OR 'tubal abortions':ti,ab,kw OR 'miscarriage':ti,ab,kw OR 'miscarriages':ti,ab,kw OR 'early pregnancy loss':ti,ab,kw OR 'early pregnancy losses':ti,ab,kw OR 'loss, early pregnancy':ti,ab,kw OR 'losses, early pregnancy':ti,ab,kw OR 'pregnancy loss, early':ti,ab,kw OR 'pregnancy losses, early':ti,ab,kw
#21.	'obstetric labor, premature'/exp OR 'labor, premature':ti,ab,kw OR 'premature labor':ti,ab,kw OR 'premature obstetric labor':ti,ab,kw OR 'preterm labor':ti,ab,kw OR 'labor, preterm':ti,ab,kw
#22.	'fetal death'/exp OR 'death, fetal':ti,ab,kw OR 'pteroylglutamic aciddeaths, fetal':ti,ab,kw OR 'fetal deaths':ti,ab,kw OR 'fetal
#23.	'stillbirth'/exp OR 'stillbirths':ti,ab,kw
#24.	'infant, small for gestational age'/exp OR 'infant, large for gestational age'/exp OR 'average gestational age at delivery':ti,ab,kw OR 'average gestational age at birth':ti,ab,kw OR 'mean gestational age at delivery':ti,ab,kw OR 'average foetal age at delivery':ti,ab,kw
#25.	'birth weight'/exp OR 'infant, low birth weight'/exp OR 'infant, extremely low birth weight'/exp OR 'infant, very low birth weight'/exp OR 'fetal macrosomia'/exp OR 'fetal macrosomias':ti,ab,kw OR 'macrosomias, fetal':ti,ab,kw OR 'macrosomia, fetal':ti,ab,kw

#26.	'apgar score'/exp OR 'respiratory distress syndrome, newborn'/exp OR 'respiratory distress syndrome, infant':ti,ab,kw OR 'infantile respiratory distress syndrome':ti,ab,kw OR 'neonatal respiratory distress syndrome':ti,ab,kw
#27.	'congenital abnormalities'/exp OR 'abnormality, congenital':ti,ab,kw OR 'congenital abnormality':ti,ab,kw OR 'abnormalities, congenital':ti,ab,kw OR 'birth defects':ti,ab,kw OR 'birth defect':ti,ab,kw OR 'defect, birth':ti,ab,kw OR 'congenital defects':ti,ab,kw OR 'congenital defect':ti,ab,kw OR 'defect, congenital':ti,ab,kw OR 'defects, congenital':ti,ab,kw OR 'deformities':ti,ab,kw OR 'deformity':ti,ab,kw OR 'fetal anomalies':ti,ab,kw OR 'anomaly, fetal':ti,ab,kw OR 'fetal anomaly':ti,ab,kw OR 'fetal malformations':ti,ab,kw OR 'fetal malformation':ti,ab,kw OR 'malformation, fetal':ti,ab,kw
#28.	'depression, postpartum'/exp OR 'postpartum depression':ti,ab,kw OR 'post-natal depression':ti,ab,kw OR 'depression, post-natal':ti,ab,kw OR 'post natal depression':ti,ab,kw OR 'post-partum depression':ti,ab,kw OR 'depression, post-partum':ti,ab,kw OR 'post partum depression':ti,ab,kw OR 'postnatal depression':ti,ab,kw OR 'depression, postnatal':ti,ab,kw OR 'postnatal dysphoria':ti,ab,kw OR 'dysphoria, postnatal':ti,ab,kw OR 'postpartum dysphoria':ti,ab,kw OR 'dysphoria, postpartum':ti,ab,kw OR 'post-partum dysphoria':ti,ab,kw OR 'dysphoria, post-partum':ti,ab,kw OR 'post partum dysphoria':ti,ab,kw OR 'post-natal dysphoria':ti,ab,kw OR 'dysphoria, post-natal':ti,ab,kw OR 'post natal dysphoria':ti,ab,kw
#29.	'perinatal death'/exp OR 'death, perinatal':ti,ab,kw OR 'deaths, perinatal':ti,ab,kw OR 'perinatal deaths':ti,ab,kw OR 'neonatal death':ti,ab,kw OR 'death, neonatal':ti,ab,kw OR 'deaths, neonatal':ti,ab,kw OR 'neonatal deaths':ti,ab,kw
#30.	'reproductive techniques, assisted'/exp OR 'assisted reproductive technique':ti,ab,kw OR 'reproductive technique, assisted':ti,ab,kw OR 'technique, assisted reproductive':ti,ab,kw OR 'techniques, assisted reproductive':ti,ab,kw OR 'assisted reproductive technics':ti,ab,kw OR 'assisted reproductive technic':ti,ab,kw OR 'reproductive technic, assisted':ti,ab,kw OR 'reproductive technics, assisted':ti,ab,kw OR 'technic, assisted reproductive':ti,ab,kw OR 'technics, assisted reproductive':ti,ab,kw OR 'assisted reproductive techniques':ti,ab,kw OR 'reproductive technology, assisted':ti,ab,kw OR 'assisted reproductive technologies':ti,ab,kw OR 'assisted reproductive technology':ti,ab,kw OR 'reproductive technologies, assisted':ti,ab,kw OR 'technologies, assisted reproductive':ti,ab,kw OR 'technology, assisted reproductive':ti,ab,kw
#31.	'biochemical pregnancy':ti,ab,kw OR 'clinical pregnancy rate':ti,ab,kw OR 'clinical gestation rate':ti,ab,kw OR 'clinical live birth rate':ti,ab,kw OR 'clinical live-birth rate':ti,ab,kw OR 'retrieved oocytes':ti,ab,kw OR 'metaphase ii rate':ti,ab,kw OR 'high-quality embryo rate':ti,ab,kw
#32.	'meta-analysis':pt OR 'meta analysis':pt OR 'systematic review':pt OR 'systematic evolution':pt

#33.	'meta-analysis as topic'/exp OR 'meta-analysis':ti,ab,kw OR 'meta analysis':ti,ab,kw OR 'systematic review':ti,ab,kw OR 'systematic evolution':ti,ab,kw
#34.	#32 OR #33
#35.	#3 OR #4 OR #5 OR #6 OR #7 OR #8 OR #11 OR #12 OR #13 OR #14 OR #15 OR #16 OR #17 OR #18 OR #19 OR #20 OR #21 OR #22 OR #23 OR #24 OR #25 OR #26 OR #27 OR #28 OR #29 OR #30 OR #31
#36.	#1 OR #2
#37.	#34 AND #35 AND #36
**Cochrane**	
ID	Search Hits
#1	MeSH descriptor: [Vitamins] explode all trees
#2	MeSH descriptor: [Folic Acid] explode all trees
#3	(Pteroylglutamic Acid):ti,ab,kw OR (Vitamin M):ti,ab,kw OR (Vitamin B9):ti,ab,kw OR (B9, Vitamin):ti,ab,kw OR (Folacin):ti,ab,kw OR (Folvite):ti,ab,kw OR (Folate)
#4	#2 OR #3
#5	MeSH descriptor: [Pregnancy Complications] explode all trees
#6	(Pregnancy Complication):ti,ab,kw OR (Complications, Pregnancy):ti,ab,kw OR (Adverse Birth Outcomes):ti,ab,kw OR (Adverse Birth Outcome):ti,ab,kw OR (Birth Outcome, Adverse):ti,ab,kw OR (Outcome, Adverse Birth):ti,ab,kw
#7	#5 OR #6
#8	MeSH descriptor: [Eclampsia] explode all trees
#9	MeSH descriptor: [Pre-Eclampsia] explode all trees
#10	(Pre Eclampsia):ti,ab,kw OR (Preeclampsia):ti,ab,kw
#11	#9 OR #10
#12	MeSH descriptor: [Hypertension, Pregnancy-Induced] explode all trees
#13	(Pregnancy-Induced Hypertension):ti,ab,kw OR (Gestational Hypertension):ti,ab,kw OR (Hypertension, Gestational):ti,ab,kw OR (Pregnancy Induced Hypertension):ti,ab,kw OR (Hypertensions, Pregnancy Induced):ti,ab,kw OR (Induced Hypertension, Pregnancy):ti,ab,kw OR (Induced Hypertensions, Pregnancy):ti,ab,kw OR (Transient Hypertension, Pregnancy):ti,ab,kw OR (Hypertension, Pregnancy Transient):ti,ab,kw OR (Pregnancy Transient Hypertension):ti,ab,kw
#14	#12 OR #13
#15	MeSH descriptor: [Diabetes, Gestational] explode all trees
#16	(Diabetes Mellitus, Gestational):ti,ab,kw OR (Gestational Diabetes Mellitus):ti,ab,kw OR (Diabetes, Pregnancy-Induced):ti,ab,kw OR (Diabetes, Pregnancy Induced):ti,ab,kw OR (Pregnancy-Induced Diabetes):ti,ab,kw OR (Gestational Diabetes):ti,ab,kw

#17	#15 OR #16
#18	MeSH descriptor: [Insulin Resistance] explode all trees
#19	(Resistance, Insulin):ti,ab,kw
#20	#18 OR #19
#21	MeSH descriptor: [Vomiting] explode all trees
#22	(Emesis):ti,ab,kw
#23	#21 OR #22
#24	MeSH descriptor: [Hyperemesis Gravidarum] explode all trees
#25	(Pernicious Vomiting of Pregnancy):ti,ab,kw OR (Pregnancy Pernicious Vomiting):ti,ab,kw
#26	#24 OR #25
#27	MeSH descriptor: [Reproductive Tract Infections] explode all trees
#28	(Infection, Reproductive Tract):ti,ab,kw OR (Infections, Reproductive Tract):ti,ab,kw OR (Reproductive Tract Infection):ti,ab,kw OR (Genital Tract Infections):ti,ab,kw OR (Genital Tract Infection):ti,ab,kw OR (Infection, Genital Tract):ti,ab,kw OR (Infections, Genital Tract):ti,ab,kw
#29	#27 OR #28
#30	MeSH descriptor: [Anemia] explode all trees
#31	(Anemias):ti,ab,kw
#32	#30 OR #31
#33	MeSH descriptor: [Fetal Membranes, Premature Rupture] explode all trees
#34	(Premature rupture of membranes):ti,ab,kw OR (Premature Rupture of Fetal Membranes):ti,ab,kw OR (Premature Rupture of Membrane (Pregnancy)):ti,ab,kw OR (Membrane Premature Rupture (Pregnancy)):ti,ab,kw OR (Membrane Premature Ruptures (Pregnancy)):ti,ab,kw OR (PROM (Pregnancy)):ti,ab,kw OR (Preterm Premature Rupture of Fetal Membranes):ti,ab,kw OR (Preterm PROM (Pregnancy)):ti,ab,kw OR (PROM, Preterm (Pregnancy)):ti,ab,kw
#35	#33 OR #34
#36	MeSH descriptor: [Abruptio Placentae] explode all trees
#37	(Placental abruption):ti,ab,kw OR (Abruption, Placental):ti,ab,kw OR (Abruptions, Placental):ti,ab,kw OR (Placental Abruptions):ti,ab,kw
#38	#36 OR #37
#39	MeSH descriptor: [Thyroid Diseases] explode all trees
#40	(Diseases, Thyroid):ti,ab,kw OR (Disease, Thyroid):ti,ab,kw OR (Thyroid Disease):ti,ab,kw
#41	#39 OR #40
#42	MeSH descriptor: [Cesarean Section] explode all trees
#43	(Cesarean Sections):ti,ab,kw OR (Abdominal Delivery):ti,ab,kw OR (C-Section (OB)):ti,ab,kw OR (C Section (OB)):ti,ab,kw OR (C-Sections (OB)):ti,ab,kw OR

	(Caesarean Section):ti,ab,kw OR (Caesarean Sections):ti,ab,kw OR (Delivery, Abdominal):ti,ab,kw OR (Abdominal Deliveries):ti,ab,kw OR (Deliveries, Abdominal):ti,ab,kw OR (Postcesarean Section):ti,ab,kw
#44	#42 OR #43
#45	(Prolongation of labour):ti,ab,kw OR (Prolonged labour):ti,ab,kw
#46	MeSH descriptor: [Postpartum Hemorrhage] explode all trees
#47	(Hemorrhage, Postpartum):ti,ab,kw OR (Delayed Postpartum Hemorrhage):ti,ab,kw OR (Hemorrhage, Delayed Postpartum):ti,ab,kw OR (Postpartum Hemorrhage, Delayed):ti,ab,kw OR (Immediate Postpartum Hemorrhage):ti,ab,kw OR (Hemorrhage, Immediate Postpartum):ti,ab,kw OR (Postpartum Hemorrhage, Immediate):ti,ab,kw
#48	#46 OR #47
#49	MeSH descriptor: [Abortion, Spontaneous] explode all trees
#50	(Spontaneous Abortions):ti,ab,kw OR (Spontaneous Abortion):ti,ab,kw OR (Abortion, Tubal):ti,ab,kw OR (Abortions, Tubal):ti,ab,kw OR (Tubal Abortion):ti,ab,kw OR (Tubal Abortions):ti,ab,kw OR (Miscarriage):ti,ab,kw OR (Miscarriages):ti,ab,kw OR (Early Pregnancy Loss):ti,ab,kw OR (Early Pregnancy Losses):ti,ab,kw OR (Loss, Early Pregnancy):ti,ab,kw OR (Losses, Early Pregnancy):ti,ab,kw OR (Pregnancy Loss, Early):ti,ab,kw OR (Pregnancy Losses, Early):ti,ab,kw
#51	#49 OR #50
#52	MeSH descriptor: [Obstetric Labor, Premature] explode all trees
#53	(Labor, Premature):ti,ab,kw OR (Premature Labor):ti,ab,kw OR (Premature Obstetric Labor):ti,ab,kw OR (Preterm Labor):ti,ab,kw OR (Labor, Preterm):ti,ab,kw
#54	#52 OR #53
#55	MeSH descriptor: [Fetal Death] explode all trees
#56	(Death, Fetal):ti,ab,kw OR (Pteroylglutamic AcidDeaths, Fetal):ti,ab,kw OR (Fetal Deaths):ti,ab,kw OR (Fetal Demise):ti,ab,kw OR (Demise, Fetal):ti,ab,kw
#57	#55 OR #56
#58	MeSH descriptor: [Stillbirth] explode all trees
#59	(Stillbirths):ti,ab,kw
#60	#58 OR #59
#61	MeSH descriptor: [Infant, Small for Gestational Age] explode all trees
#62	MeSH descriptor: [Infant, Large for Gestational Age] explode all trees
#63	(Average gestational age at delivery):ti,ab,kw OR (Average gestational age at birth):ti,ab,kw OR (Mean gestational age at delivery):ti,ab,kw OR (Average foetal age at delivery):ti,ab,kw
#64	#61 OR #62 OR #63
#65	MeSH descriptor: [Birth Weight] explode all trees
#66	MeSH descriptor: [Infant, Low Birth Weight] explode all trees
#67	MeSH descriptor: [Infant, Extremely Low Birth Weight] explode all trees

#68	MeSH descriptor: [Infant, Very Low Birth Weight] explode all trees
#69	MeSH descriptor: [Fetal Macrosomia] explode all trees
#70	(Fetal Macrosomias):ti,ab,kw OR (Macrosomias, Fetal):ti,ab,kw OR (Macrosomia, Fetal):ti,ab,kw
#71	#69 OR #70
#72	#65 OR #66 OR #67 OR #68 OR #71
#73	MeSH descriptor: [Apgar Score] explode all trees
#74	MeSH descriptor: [Respiratory Distress Syndrome, Newborn] explode all trees
#75	(Respiratory Distress Syndrome, Infant):ti,ab,kw OR (Infantile Respiratory Distress Syndrome):ti,ab,kw OR (Neonatal Respiratory Distress Syndrome):ti,ab,kw
#76	#74 OR #75
#77	MeSH descriptor: [Congenital Abnormalities] explode all trees
#78	(Abnormality, Congenital):ti,ab,kw OR (Congenital Abnormality):ti,ab,kw OR (Abnormalities, Congenital):ti,ab,kw OR (Birth Defects):ti,ab,kw OR (Birth Defect):ti,ab,kw OR (Defect, Birth):ti,ab,kw OR (Congenital Defects):ti,ab,kw OR (Congenital Defect):ti,ab,kw OR (Defect, Congenital):ti,ab,kw OR (Defects, Congenital):ti,ab,kw OR (Deformities):ti,ab,kw OR (Deformity):ti,ab,kw OR (Fetal Anomalies):ti,ab,kw OR (Anomaly, Fetal):ti,ab,kw OR (Fetal Anomaly):ti,ab,kw OR (Fetal Malformations):ti,ab,kw OR (Fetal Malformation):ti,ab,kw OR (Malformation, Fetal):ti,ab,kw
#79	#77 OR #78
#80	MeSH descriptor: [Depression, Postpartum] explode all trees
#81	(Postpartum Depression):ti,ab,kw OR (Post-Natal Depression):ti,ab,kw OR (Depression, Post-Natal):ti,ab,kw OR (Post Natal Depression):ti,ab,kw OR (Post-Partum Depression):ti,ab,kw OR (Depression, Post-Partum):ti,ab,kw OR (Post Partum Depression):ti,ab,kw OR (Postnatal Depression):ti,ab,kw OR (Depression, Postnatal):ti,ab,kw OR (Postnatal Dysphoria):ti,ab,kw OR (Dysphoria, Postnatal):ti,ab,kw OR (Postpartum Dysphoria):ti,ab,kw OR (Dysphoria, Postpartum):ti,ab,kw OR (Post-Partum Dysphoria):ti,ab,kw OR (Dysphoria, Post-Partum):ti,ab,kw OR (Post Partum Dysphoria):ti,ab,kw OR (Post-Natal Dysphoria):ti,ab,kw OR (Dysphoria, Post-Natal):ti,ab,kw OR (Post Natal Dysphoria):ti,ab,kw
#82	#80 OR #81
#83	MeSH descriptor: [Perinatal Death] explode all trees
#84	(Death, Perinatal):ti,ab,kw OR (Deaths, Perinatal):ti,ab,kw OR (Perinatal Deaths):ti,ab,kw OR (Neonatal Death):ti,ab,kw OR (Death, Neonatal):ti,ab,kw OR (Deaths, Neonatal):ti,ab,kw OR (Neonatal Deaths):ti,ab,kw
#85	#83 OR #84
#86	MeSH descriptor: [Reproductive Techniques, Assisted] explode all trees
#87	(Assisted Reproductive Technique):ti,ab,kw OR (Assisted Reproductive Technique):ti,ab,kw OR (Reproductive Technique, Assisted):ti,ab,kw OR

	(Technique, Assisted Reproductive):ti,ab,kw OR (Techniques, Assisted Reproductive):ti,ab,kw OR (Assisted Reproductive Technics):ti,ab,kw OR (Assisted Reproductive Technic):ti,ab,kw OR (Reproductive Technic, Assisted):ti,ab,kw OR (Reproductive Technics, Assisted):ti,ab,kw OR (Technic, Assisted Reproductive):ti,ab,kw OR (Technics, Assisted Reproductive):ti,ab,kw OR (Assisted Reproductive Techniques):ti,ab,kw OR (Reproductive Technology, Assisted):ti,ab,kw OR (Assisted Reproductive Technologies):ti,ab,kw OR (Assisted Reproductive Technology):ti,ab,kw OR (Reproductive Technologies, Assisted):ti,ab,kw OR (Technologies, Assisted Reproductive):ti,ab,kw OR (Technology, Assisted Reproductive):ti,ab,kw
#88	#86 OR #87
#89	(Biochemical pregnancy):ti,ab,kw OR (Clinical pregnancy rate):ti,ab,kw OR (Clinical gestation rate):ti,ab,kw OR (Clinical live birth rate):ti,ab,kw OR (Clinical live-birth rate):ti,ab,kw OR (Retrieved oocytes):ti,ab,kw OR (Metaphase II rate):ti,ab,kw OR (High-quality embryo rate):ti,ab,kw
#90	#88 OR #89
#91	(meta-analysis):pt OR (meta analysis):pt OR (systematic review):pt OR (systematic evolution):pt
#92	MeSH descriptor: [Meta-Analysis as Topic] explode all trees
#93	(meta-analysis):ti,ab,kw OR (meta analysis):ti,ab,kw OR (systematic review):ti,ab,kw OR (systematic evolution):ti,ab,kw
#94	#91 OR #92 OR #93
#95	(#1 OR #4) AND (#7 OR #8 OR #11 OR #14 OR #17 OR #20 OR #23 OR #26 OR #29 OR #32 OR #35 OR #38 OR #41 OR #44 OR #45 OR #48 OR #51 OR #54 OR #57 OR #60 OR #64 OR #72 OR #73 OR #76 OR #79 OR #82 OR #85 OR #90) AND #94

Table S2 Quality Assessment of included studies using AMSTAR 2 Tool

	1	2	3	4	5	6	7	8	9	10	11	12	13	14	15	16	AMSTAR-2
Pejcic 2024	Yes	Yes	Yes	Yes	Yes	Yes	Yes	Yes	Yes	No	Yes	Yes	Yes	Yes	Yes	Yes	High
Aguilar-Cordero 2020	Yes	Yes	Yes	Partial yes	No	Yes	Partial yes	Yes	Yes	No	Yes	Yes	Yes	Yes	Yes	Yes	Moderate
Centeno 2024	Yes	Yes	Yes	Yes	Yes	Yes	Partial yes	Yes	Yes	Yes	Yes	Yes	Yes	Yes	Yes	Yes	Moderate
Cozzolino 2020	Yes	Yes	Yes	Yes	Yes	Yes	Partial yes	Yes	Yes	No	Yes	Yes	Yes	Yes	Yes	Yes	Moderate
De-Regil 2012	Yes	No	Yes	Yes	Yes	Yes	Yes	Yes	Yes	No	Yes	No	Yes	Yes	Yes	Yes	Moderate
Hu 2022	Yes	Yes	Yes	Yes	Yes	Yes	Partial yes	Yes	Yes	No	Yes	Yes	Yes	Yes	Yes	Yes	Moderate
Lima 2022	Yes	Yes	Yes	Partial yes	Yes	Yes	Partial yes	Yes	Yes	No	Yes	Yes	Yes	Yes	Yes	Yes	Moderate
Quotah 2024	Yes	Yes	Yes	Yes	Yes	Yes	Partial yes	Yes	Yes	No	Yes	No	Yes	Yes	Yes	Yes	Moderate
Rogne 2017	Yes	Yes	No	Yes	Yes	Yes	Yes	Yes	Yes	No	Yes	Yes	Yes	Yes	Yes	Yes	Moderate
Sharifipour 2020	Yes	Yes	Yes	Partial yes	No	Yes	Partial yes	Yes	Yes	No	Yes	Yes	Yes	Yes	Yes	Yes	Moderate
Tous 2020	Yes	Yes	No	Yes	Yes	Yes	Partial yes	Yes	Yes	No	Yes	Yes	Yes	Yes	Yes	Yes	Moderate

Xu 2025	Yes	Yes	Yes	Partial yes	Yes	Yes	Partial yes	Yes	Yes	No	Yes	Yes	Yes	Yes	Yes	Yes	Moderate
Bi 2018	Yes	No	Yes	Partial yes	Yes	Yes	Partial yes	Yes	Yes	No	Yes	Yes	Yes	Yes	Yes	Yes	Low
Chen 2017	Yes	No	Yes	Partial yes	No	Yes	Partial yes	Yes	Yes	No	Yes	Yes	Yes	Yes	Yes	Yes	Low
X. Chen 2022	Yes	No	No	Partial yes	Yes	Yes	Partial yes	Yes	Yes	No	Yes	Yes	Yes	Yes	Yes	Yes	Low
C. Chen 2022	Yes	No	No	Partial yes	Yes	Yes	Partial yes	Yes	Yes	No	Yes	Yes	Yes	Yes	Yes	Yes	Low
Chu 2018	Yes	No	Yes	Yes	Yes	Yes	Partial yes	Yes	Yes	No	Yes	Yes	Yes	Yes	Yes	Yes	Low
Cui 2024	Yes	No	Yes	Partial yes	Yes	Yes	Partial yes	Yes	Yes	No	Yes	Yes	Yes	Yes	Yes	Yes	Low
De-Regil 2016	Yes	No	Yes	Yes	Yes	Yes	Yes	Yes	Yes	Yes	Yes	No	Yes	Yes	Yes	Yes	Low
He 2022	Yes	No	Yes	Yes	Yes	Yes	Partial yes	Yes	Yes	No	Yes	Yes	Yes	Yes	Yes	Yes	Low
Iliuta 2022	Yes	Yes	Yes	Partial yes	No	No	Partial yes	Yes	Yes	No	Yes	Yes	Yes	Yes	No	Yes	Low
Jin 2022	Partial yes	No	Yes	Partial yes	Yes	Yes	Yes	Yes	Yes	No	Yes	Yes	Yes	Yes	Yes	Yes	Low
Keats 2021	Yes	No	No	Yes	Yes	Yes	Yes	Yes	Yes	Yes	Yes	Yes	Yes	Yes	Yes	Yes	Low
Lassi 2013	Yes	No	Yes	Yes	Yes	Yes	Yes	Yes	Yes	No	Yes	Yes	Yes	Yes	Yes	Yes	Low

Lian 2021	Yes	No	Yes	Yes	Yes	Yes	Partial yes	Yes	Yes	No	Yes	Yes	Yes	Yes	Yes	Yes	Low
Liu 2022	Yes	No	Yes	Yes	Yes	Yes	Partial yes	Yes	Yes	No	Yes	Yes	Yes	Yes	Yes	Yes	Low
Lv 2016	Yes	No	No	Partial yes	Yes	Yes	Partial yes	Yes	Yes	No	Yes	Yes	Yes	Yes	Yes	Yes	Low
Mardali 2021	Yes	No	Yes	Yes	Yes	No	Partial yes	Yes	Yes	No	Yes	Yes	Yes	Yes	Yes	Yes	Low
Maugeri 2019	Yes	No	Yes	Yes	Yes	Yes	Partial yes	Yes	Yes	No	Yes	Yes	Yes	Yes	Yes	Yes	Low
Moslehi 2022	Yes	No	Yes	Yes	Yes	Yes	Partial yes	Yes	Yes	No	Yes	Yes	Yes	Yes	Yes	Yes	Low
Nargesi 2018	Yes	No	No	Yes	Yes	Yes	Partial yes	Yes	Yes	No	Yes	Yes	Yes	Yes	Yes	Yes	Low
Palacios 2024	Yes	No	Yes	Yes	Yes	Yes	Yes	Yes	Yes	Yes	Yes	Yes	Yes	Yes	Yes	Yes	Low
Rumbold 2015	Yes	No	Yes	Yes	Yes	Yes	Yes	Yes	Yes	No	Yes	No	Yes	Yes	Yes	Yes	Low
Tamblyn 2022	Yes	Yes	Yes	Yes	Yes	Yes	Partial yes	Yes	Yes	No	Yes	Partial yes	Yes	Yes	No	Yes	Low
Wang 2018	Yes	No	Yes	Yes	Yes	Yes	Partial yes	Yes	Yes	No	Yes	Yes	Yes	Yes	Yes	Yes	Low
Wei 2013	Yes	No	No	Yes	Yes	Yes	Partial yes	Yes	Yes	No	Yes	Yes	Yes	Yes	Yes	Yes	Low
Xu 2023	Yes	No	Yes	Partial yes	Yes	Yes	Partial yes	Yes	Yes	No	Yes	Yes	Yes	Yes	Yes	Yes	Low

Yang 2016	Yes	No	Yes	Yes	Yes	Yes	Partial yes	Yes	Yes	No	Yes	Yes	Yes	Yes	Yes	Yes	Low
You 2024	Yes	Yes	Yes	Yes	Yes	Yes	No	Yes	Yes	No	Yes	Yes	Yes	Yes	Yes	Yes	Low
Yuan 2024	Yes	No	Yes	Partial yes	Yes	Yes	Partial yes	Yes	Yes	No	Yes	Yes	Yes	Yes	Yes	Yes	Low
Zhang 2015	Yes	No	Yes	Partial yes	Yes	Yes	Partial yes	Yes	Yes	No	Yes	Yes	Yes	Yes	Yes	Yes	Low
Zhao 2019	Yes	No	Yes	Partial yes	Yes	Yes	Partial yes	Yes	Yes	No	Yes	No	Yes	Yes	Yes	Yes	Low
Zhou 2017	Yes	No	Yes	Yes	Yes	Yes	Partial yes	Yes	Yes	No	Yes	Yes	Yes	Yes	Yes	Yes	Low
Zhou 2022	Yes	No	Yes	Yes	Yes	Yes	Partial yes	Yes	Yes	No	Yes	No	Yes	Yes	Yes	Yes	Low
Zhou 2023	Yes	No	Yes	Yes	Yes	Yes	Partial yes	Yes	Yes	Yes	Yes	Yes	Yes	Yes	Yes	Yes	Low
Aghajafari 2013	Yes	No	Yes	Yes	Yes	Yes	Partial yes	Yes	No	No	Yes	Yes	Yes	Yes	Yes	Yes	Very Low
Amraei 2018	Yes	No	Yes	Yes	Yes	Yes	Partial yes	Yes	No	No	Yes	Yes	Yes	Yes	Yes	Yes	Very Low
Boskabadi 2025	Yes	No	Yes	Yes	No	Yes	No	Yes	Yes	No	Yes	Yes	Yes	Yes	Yes	Yes	Very Low
Chan 2021	Yes	Yes	Yes	Partial yes	Yes	Yes	Partial yes	Yes	Yes	No	Yes	No	No	Yes	No	Yes	Very Low
Fatima 2022	Yes	No	Yes	Yes	Yes	Yes	No	Yes	Yes	No	Yes	Yes	Yes	Yes	Yes	Yes	Very Low

Hu 2018	Yes	No	Yes	Yes	Yes	Yes	Partial yes	Yes	No	No	Yes	Yes	Yes	Yes	Yes	Yes	Very Low
Hyppönen 2013	Yes	No	No	Yes	Yes	Yes	Partial yes	Yes	No	No	Yes	Yes	Yes	Yes	No	Yes	Very Low
Lassi 2020	Yes	No	Yes	Yes	Yes	Yes	No	Yes	Yes	No	Yes	No	No	Yes	No	Yes	Very Low
Lu 2016	Yes	No	Yes	Yes	Yes	Yes	No	Yes	Yes	No	Yes	Yes	Yes	Yes	Yes	Yes	Very Low
McCauley 2015	Yes	No	Yes	Yes	Yes	Yes	Yes	Yes	Yes	No	Yes	Yes	Yes	Yes	No	Yes	Very Low
Milajerdi 2021	Yes	Yes	Yes	Partial yes	No	Yes	Partial yes	Yes	No	No	Yes	Yes	Yes	Yes	No	Yes	Very Low
Oh 2020	Yes	No	No	Yes	No	No	Partial yes	Yes	Yes	No	Yes	Yes	Yes	Yes	No	Yes	Very Low
Poel 2012	Yes	No	Yes	Yes	No	No	No	Yes	No	No	Yes	No	Yes	Yes	Yes	Yes	Very Low
Qin 2016	Yes	No	Yes	Yes	Yes	No	No	Yes	Yes	No	Yes	Yes	Yes	Yes	Yes	Yes	Very Low
Shen 2019	Yes	No	Yes	Partial yes	No	No	No	Yes	No	No	Yes	No	Yes	Yes	No	No	Very Low
Thorne-Lyman 2012	Yes	No	Yes	Yes	No	No	Partial yes	Yes	Yes	No	Yes	Yes	No	Yes	Yes	Yes	Very Low
Tripathi 2019	Yes	No	Yes	Yes	Yes	Yes	Partial yes	Yes	No	No	Yes	Yes	Yes	Yes	Yes	Yes	Very Low
Wang 2020	Yes	No	No	Yes	Yes	Yes	Partial yes	Yes	No	No	Yes	Yes	Yes	Yes	Yes	Yes	Very Low

Wang 2023	Yes	No	Yes	Partial yes	Yes	Yes	No	Yes	Yes	No	Yes	Yes	Yes	Yes	Yes	Yes	Very Low
Zhang 2017	Yes	No	Yes	Yes	Yes	Yes	Partial yes	Yes	Yes	No	Yes	Yes	Yes	Yes	No	Yes	Very Low

1. Did the research questions and inclusion criteria for the review include the components of PICO? 2. Did the report of the review contain an explicit statement that the review methods were established prior to the conduct of the review and did the report justify any significant deviations from the protocol? 3. Did the review authors explain their selection of the study designs for inclusion in the review? 4. Did the review authors use a comprehensive literature search strategy? 5. Did the review authors perform study selection in duplicate? 6. Did the review authors perform data extraction in duplicate? 7. Did the review authors provide a list of excluded studies and justify the exclusions? 8. Did the review authors describe the included studies in adequate detail? 9. Did the review authors use a satisfactory technique for assessing the risk of bias (RoB) in individual studies that were included in the review? 10. Did the review authors report on the sources of funding for the studies included in the review? 11. If meta-analysis was performed did the review authors use appropriate methods for statistical combination of results? 12. If meta-analysis was performed, did the review authors assess the potential impact of RoB in individual studies on the results of the meta-analysis or other evidence synthesis? 13. Did the review authors account for RoB in individual studies when interpreting/discussing the results of the review? 14. Did the review authors provide a satisfactory explanation for, and discussion of, any heterogeneity observed in the results of the review? 15. If they performed quantitative synthesis did the review authors carry out an adequate investigation of publication bias (small study bias) and discuss its likely impact on the results of the review? 16. Did the review authors report any potential sources of conflict of interest, including any funding they received for conducting the review?

Table S3 Overlapping and non-overlapping associations by corrected covered area (CCA) calculations

Index	Author, Year	Quality	Vitamin condition	Outcome	Data analysis	CCA	Decision
1	Xu 2025	Moderate	Vitamin D, low level < 20 vs high level ≥ 20 (ng/mL)	Outcomes of assisted reproductive technology (continuing pregnancy rate)	MA (5)	28.13%	Yes
	Iliuta 2022	Low			MA (11)		No
	Cozzolino 2020	Moderate			MA (9)		No
2	Xu 2025	Moderate	Vitamin D, low level < 30 vs high level ≥ 30 (ng/mL)	Outcomes of assisted reproductive technology (continuing pregnancy rate)	MA (5)	27.27%	Yes
	Iliuta 2022	Low			MA (9)		No
3	Iliuta 2022	Low	Vitamin D, deficiency vs normal	Outcomes of assisted reproductive technology (continuing pregnancy rate)	MA (11)	33.33%	Yes
	Cozzolino 2020	Moderate			MA (5)		No
4	Xu 2025	Moderate	Vitamin D, low level < 20 vs high level ≥ 20 (ng/mL)	Outcomes of assisted reproductive technology (live birth rate)	MA (7)	26.19%	Yes
	Iliuta 2022	Low			MA (6)		No
	Cozzolino 2020	Moderate			MA (9)		No
	Lv 2016	Low			MA (3)		No
5	Xu 2025	Moderate	Vitamin D, low level < 30 vs high level ≥ 30 (ng/mL)	Outcomes of assisted reproductive technology (live birth rate)	MA (6)	31.82%	Yes
	Iliuta 2022	Low			MA (5)		No

	Chu 2018	Low			MA (7)		No
6	Iliuta 2022	Low	Vitamin D, deficiency vs normal	Outcomes of assisted reproductive technology (live birth rate)	MA (6)	28.57%	Yes
	Zhao 2019	Low			MA (3)		No
7	Xu 2025	Moderate	Vitamin D, low level < 20 vs high level ≥ 20 (ng/mL)	Outcomes of assisted reproductive technology (clinical pregnancy rate)	MA (21)	25.00%	Yes
	Iliuta 2022	Low			MA (14)		No
	Cozzolino 2020	Moderate			MA (9)		No
	Lv 2016	Low			MA (5)		No
8	Xu 2025	Moderate	Vitamin D, low level < 30 vs high level ≥ 30 (ng/mL)	Outcomes of assisted reproductive technology (clinical pregnancy rate)	MA (16)	34.72%	Yes
	Iliuta 2022	Low			MA (14)		No
	Cozzolino 2020	Moderate			MA (8)		No
	Chu 2018	Low			MA (11)		No
9	Cozzolino 2020	Moderate	Vitamin D, deficiency vs normal	Outcomes of assisted reproductive technology (clinical pregnancy rate)	MA (4)	18.18%	No
	Zhao 2019	Low			MA (9)		Yes
10	Xu 2025	Moderate	Vitamin D, low level < 20 vs high level ≥ 20 (ng/mL)	Outcomes of assisted reproductive technology (miscarriage rate)	MA (5)	25.00%	Yes
	Iliuta 2022	Low			MA (11)		No

	Cozzolino 2020	Moderate			MA (8)		No
11	Iliuta 2022	Low	Vitamin D, low level < 30 vs high level ≥ 30 (ng/mL)	Outcomes of assisted reproductive technology (miscarriage rate)	MA (9)	37.50%	Yes
	Cozzolino 2020	Moderate			MA (6)		No
	Chu 2018	Low			MA (6)		No
12	Iliuta 2022	Low	Vitamin D, deficiency vs normal	Outcomes of assisted reproductive technology (miscarriage rate)	MA (11)	33.33%	Yes
	Cozzolino 2020	Moderate			MA (5)		No
13	Xu 2025	Moderate	Vitamin D, low level < 20 vs high level ≥ 20 (ng/mL)	Outcomes of assisted reproductive technology (biochemical pregnancy rate)	MA (3)	11.11%	Yes
	Iliuta 2022	Low			MA (7)		No
14	Xu 2025	Moderate	Vitamin D, low level < 30 vs high level ≥ 30 (ng/mL)	Outcomes of assisted reproductive technology (biochemical pregnancy rate)	MA (3)	22.22%	Yes
	Iliuta 2022	Low			MA (5)		No
	Chu 2018	Low			MA (5)		No
15	Xu 2025	Moderate	Vitamin D, low level < 30 vs high level ≥ 30 (ng/mL)	Outcomes of assisted reproductive technology (implantation rate)	MA (5)	37.5%	Yes
	Iliuta 2022	Low			MA (6)		No
16	Palacios 2024	Low	Vitamin D, Supplementation	Neonatal outcome (low birth weight infants)	MA (3)	13.33%	No
	Liu 2022	Low			MA (10)		Yes

	Bi 2018	Low			MA (4)		No
	De-Regil 2016	Low			MA (3)		No
	De-Regil 2012	Moderate			MA (3)		No
17	Zhang 2015	Low	Vitamin D, low level < 20 vs high level ≥ 20 (ng/mL)	Gestational diabetes	MA (20)	42.86%	Yes
	Wei 2013	Low			MA (10)		No
18	Moslehi 24 2022	Low	Vitamin D, level, WMD	Gestational diabetes	MA (7)	9.52%	Yes
	Zhang 67 2015	Low			MA (20)		Yes
19	Liu 2022	Low	Vitamin D, Supplementation	Neonatal outcome (birth length)	MA (20)	15.91%	Yes
	Maugeri 2019	Low			MA (5)		No
	De-Regil 2016	Low			MA (4)		No
20	Palacios 2024	Low	Vitamin D, Supplementation	Neonatal outcome (stillbirth)	MA (3)	27.08%	No
	Liu 2022	Low			MA (13)		Yes
	Bi 2018	Low			MA (10)		No
	De-Regil 2016	Low			MA (3)		No
21	Liu 2022	Low	Vitamin D, Supplementation	Neonatal outcome (birth weight)	MA (33)	20.27%	Yes

	Maugeri 2019	Low			MA (10)		No
	Bi 2018	Low			MA (17)		No
	De-Regil 2016	Low			MA (5)		No
	De-Regil 2012	Moderate			MA (3)		No
22	Liu 2022	Low	Vitamin D, Supplementation	Neonatal outcome (birth head circumference)	MA (18)	15.00%	Yes
	Maugeri 2019	Low			MA (4)		No
	De-Regil 2016	Low			MA (4)		No
23	Tous 2020	Moderate	Vitamin D, low level < 20 vs high level ≥ 20 (ng/mL)	Neonatal outcome (small for gestational age)	MA (19)	20.83%	Yes
	Chen 2017	Low			MA (9)		No
	Wei 2013	Low			MA (6)		No
24	Tous 2020	Moderate	Vitamin D, low level < 30 vs high level ≥ 30 (ng/mL)	Neonatal outcome (small for gestational age)	MA (7)	20.00%	Yes
	Wei 2013	Low			MA (5)		No
25	Liu 2022	Low	Vitamin D, Supplementation	Neonatal outcome (small for gestational age)	MA (10)	45.45%	Yes
	Bi 2018	Low			MA (6)		No
26	Lian 2021（1）	Low	Vitamin D, low level < 20 vs high level ≥ 20 (ng/mL)	Neonatal outcome (preterm birth)	MA (9)	16.25%	No

	Lian 2021（2）	Low			MA (11)		No
	Lian 2021（3）	Low			MA (9)		No
	Aguilar-Cordero 2020	Moderate			MA (9)		No
	Tous 2020	Moderate			MA (21)		Yes
	Zhou 2017	Low			MA (16)		No
	Wei 2013	Low			MA (4)		No
27	Aguilar-Cordero 2020	Moderate	Vitamin D, low level < 30 vs high level ≥ 30 (ng/mL)	Neonatal outcome (preterm birth)	MA (7)	17.39%	No
	Tous 2020	Moderate			MA (10)		Yes
	Zhou 2017	Low			MA (4)		No
	Wei 2013	Low			MA (15)		No
28	Palacios 2024	Low	Vitamin D, Supplementation	Neonatal outcome (preterm birth)	MA (3)	16.67%	No
	Jin 2022（1）	Low			MA (6)		No
	Jin 2022（2）	Low			MA (10)		No
	Liu 2022	Low			MA (21)		Yes
	Bi 2018	Low			MA (11)		No

	Zhou 2017	Low			MA (6)		No
	De-Regil 2016	Low			MA (3)		No
29	Hu 2022	Moderate	Vitamin D, low level < 20 vs high level ≥ 20 (ng/mL)	Pre-eclampsia	MA (19)	22.00%	Yes
	Aguilar-Cordero 2020	Moderate			MA (11)		No
	Wei 2013	Low			MA (6)		No
30	Hu 2022	Moderate	Vitamin D, low level < 30 vs high level ≥ 30 (ng/mL)	Pre-eclampsia	MA (14)	18.18%	Yes
	Aguilar-Cordero 2020	Moderate			MA (11)		No
	Wei 2013	Low			MA (5)		No
31	Chen 2022	Low	Vitamin B12, deficiency	Gestational diabetes	MA (6)	11.54%	No
	He 2022（1）	Low			MA (6)		Yes
	He 2022（2）	Low			MA (4)		Yes
32	Chen 2022	Low	Vitamin B12, level	Gestational diabetes	MA (12)	58.33%	Yes
	Wang 2021	Low			MA (7)		No
33	Pejcic 2024	High	Vitamin C, Supplementation	Premature rupture of membranes (preterm labour)	MA (4)	28.57%	Yes
	Rumbold 2015	Low			MA (5)		No

34	Xu<br>2023	Low	Folic acid, level	Gestational diabetes	MA (17)	47.06%	Yes
	Wang<br>2021	Low			MA (8)		No

CCA：corrected covered area

Table S4 List of excluded studies

No	Author	Year	Title	Reason for exclusion
1.	Aghajafari	2013	Aghajafari F, Nagulesapillai T, Ronksley P E, et al. Association between maternal serum 25-hydroxyvitamin D level and pregnancy and neonatal outcomes: systematic review and meta-analysis of observational studies.[J]. BMJ (Clinical research ed.), 2013, 346(7902): f1169.	Critical low quality
2.	Aguilar-Cordero	2020	Aguilar-Cordero M J, Lasserrot-Cuadrado A, Mur-Villar N, et al. Vitamin D, preeclampsia and prematurity: A systematic review and meta-analysis of observational and interventional studies.[J]. Midwifery, 2020, 87: 102707.	Study overlap
3.	Amini	2024	Amini S, Sheykhon A A, Abiri B, et al. Maternal Dietary Intakes and Risk of Spontaneous Abortion: A Systematic Review[J]. Current Nutrition and Food Science, 2024, 20(6): 716-725.	Further findings do not meet the inclusion criteria
4.	Amraei	2018	Amraei M, Mohamadpour S, Sayehmiri K, et al. Effects of Vitamin D Deficiency on Incidence Risk of Gestational Diabetes Mellitus: A Systematic Review and Meta-analysis.[J]. Frontiers in endocrinology, 2018, 9(FEB): 7.	Critical low quality
5.	Bi	2018	Bi W G, Nuyt A M, Weiler H, et al. Association Between Vitamin D Supplementation During Pregnancy and Offspring Growth, Morbidity, and Mortality: A Systematic Review and Meta-analysis.[J]. JAMA pediatrics, 2018, 172(7): 635-645.	Study overlap
6.	Boskabadi	2025	Boskabadi H, Zakerihamidi M, Mehrad-Majd H, et al. Evaluation of vitamin D in the diagnosis of infants with respiratory distress, the clinical value: A systematic review and meta-analysis[J]. Paediatric Respiratory Reviews, 2025, 53: 44-54.	Critical low quality
7.	Chan	2021	Chan K Y, Wong M M H, Pang S S H, et al. Dietary supplementation for gestational diabetes prevention and management: a meta-analysis of randomized controlled trials.[J]. Archives of gynecology and obstetrics, 2021, 303(6): 1381-1391.	Critical low quality

8.	Chen	2017	Chen Y, Zhu B, Wu X, et al. Association between maternal Vitamin D deficiency and small for gestational age: Evidence from a meta-analysis of prospective cohort studies[J]. BMJ Open, 2017, 7(8).	Study overlap
9.	Chu	2018	Chu J, Gallos I, Tobias A, et al. Vitamin D and assisted reproductive treatment outcome: a systematic review and meta-analysis.[J]. Human reproduction (Oxford, England), 2018, 33(1): 65-80.	Study overlap
10.	De-Regil	2016	De-Regil L M, Palacios C, Lombardo L K, et al. Vitamin D supplementation for women during pregnancy.[J]. The Cochrane database of systematic reviews, 2016(1): CD008873.	Study overlap
11.	De-Regil	2012	De-Regil L M, Palacios C, Ansary A, et al. Vitamin D supplementation for women during pregnancy.[J]. The Cochrane database of systematic reviews, 2012, 2(2): CD008873.	Study overlap
12.	Fatima	2022	Fatima K, Asif M, Nihal K, et al. Association between vitamin D levels in early pregnancy and gestational diabetes mellitus: A systematic review and meta-analysis.[J]. Journal of family medicine and primary care, 2022, 11(9): 5569-5580.	Critical low quality
13.	Hu	2018	Hu L, Zhang Y, Wang X, et al. Maternal Vitamin D Status and Risk of Gestational Diabetes: a Meta-Analysis.[J]. Cellular physiology and biochemistry : international journal of experimental cellular physiology, biochemistry, and pharmacology, 2018, 45(1): 291-300.	Critical low quality
14.	Hyppönen	2013	Hyppönen E, Cavadino A, Williams D, et al. Vitamin D and pre-eclampsia: original data, systematic review and meta-analysis.[J]. Annals of nutrition & metabolism, 2013, 63(4): 331-340.	Critical low quality
15.	Lassi	2020	Lassi Z S, Kedzior S G, Tariq W, et al. Effects of Preconception Care and Periconception Interventions on Maternal Nutritional Status and Birth Outcomes in Low- and Middle-Income Countries: A Systematic Review.[J]. Nutrients, 2020, 12(3).	Critical low quality
16.	Li	2022	Li N, Jiang J, Guo L. Effects of maternal folate and vitamin B12 on gestational diabetes mellitus: a dose-response meta-analysis of observational studies.[J]. European journal of clinical nutrition, 2022, 76(11): 1502-1512.	Further findings do not meet the inclusion criteria

17.	Lian	2021	Lian R H, Qi P A, Yuan T, et al. Systematic review and meta-Analysis of vitamin D deficiency in different pregnancy on preterm birth: Deficiency in middle pregnancy might be at risk[J]. Medicine (United States), 2021, 100(24): E26303.	Study overlap
18.	Lu	2016	Lu M, Xu Y, Lv L, et al. Association between vitamin D status and the risk of gestational diabetes mellitus: a meta-analysis.[J]. Archives of gynecology and obstetrics, 2016, 293(5): 959-966.	Critical low quality
19.	Lv	2016	Lv S S, Wang J Y, Wang X Q, et al. Serum vitamin D status and in vitro fertilization outcomes: a systematic review and meta-analysis.[J]. Archives of gynecology and obstetrics, 2016, 293(6): 1339-1345.	Study overlap
20.	Maugeri	2019	Maugeri A, Barchitta M, Blanco I, et al. Effects of Vitamin D supplementation during pregnancy on birth size: A systematic review and meta-analysis of randomized controlled trials[J]. Nutrients, 2019, 11(2).	Study overlap
21.	McCauley	2015	McCauley M E, van den Broek N, Dou L, et al. Vitamin A supplementation during pregnancy for maternal and newborn outcomes.[J]. The Cochrane database of systematic reviews, 2015, 2015(10): CD008666.	Critical low quality
22.	Milajerdi	2021	Milajerdi A, Abbasi F, Mousavi S M, et al. Maternal vitamin D status and risk of gestational diabetes mellitus: A systematic review and meta-analysis of prospective cohort studies.[J]. Clinical nutrition, 2021, 40(5): 2576-2586.	Critical low quality
23.	Moghib	2024	Moghib K, Ghanm T I, Abunamoos A, et al. Efficacy of vitamin D supplementation on the incidence of preeclampsia: a systematic review and meta-analysis.[J]. BMC pregnancy and childbirth, 2024, 24(1): 852.	Further findings do not meet the inclusion criteria
24.	Oh	2020	Oh C, Keats E C, Bhutta Z A. Vitamin and Mineral Supplementation During Pregnancy on Maternal, Birth, Child Health and Development Outcomes in Low- and Middle-Income Countries: A Systematic Review and Meta-Analysis.[J]. Nutrients, 2020, 12(2).	Critical low quality
25.	Palacios	2024	Palacios C, Kostiuk L L, Cuthbert A, et al. Vitamin D supplementation for women during pregnancy.[J]. The Cochrane database of systematic reviews, 2024, 7(7): CD008873.	Study overlap

26.	Poel	2012	Poel Y H M, Hummel P, Lips P, et al. Vitamin D and gestational diabetes: a systematic review and meta-analysis.[J]. European journal of internal medicine, 2012, 23(5): 465-469.	Critical low quality
27.	Qin	2016	Qin L L, Lu F G, Yang S H, et al. Does maternal Vitamin D deficiency increase the risk of preterm birth: A meta-analysis of observational studies[J]. Nutrients, 2016, 8(5).	Critical low quality
28.	Quotah	2024	Quotah O F, Andreeva D, Nowak K G, et al. Interventions in preconception and pregnant women at risk of gestational diabetes; a systematic review and meta-analysis of randomised controlled trials.[J]. Diabetology & metabolic syndrome, 2024, 16(1): 8.	Study overlap
29.	Rouhani	2023	Rouhani P, Mokhtari E, Lotfi K, et al. The association between circulating 25-hydroxyvitamin D levels and preeclampsia: a systematic review and dose-response meta-analysis of epidemiologic studies with GRADE assessment[J]. Nutrition Reviews, 2023, 81(10): 1267-1289.	Further findings do not meet the inclusion criteria
30.	Shen	2019	Shen C, Wang L, Wu X, et al. The relationship between Vitamin D and IVF: A systematic review and meta-analysis[J]. Clinical and Experimental Obstetrics and Gynecology, 2019, 46(1): 12-15.	Critical low quality
31.	Tesfay	2023	Tesfay N, Hailu G, Habtetsion M, et al. Birth prevalence and risk factors of neural tube defects in Ethiopia: a systematic review and meta-analysis[J]. BMJ Open, 2023, 13(11).	Further findings do not meet the inclusion criteria
32.	Thorne-Lyman	2012	Thorne-Lyman A, Fawzi W W. Vitamin D during pregnancy and maternal, neonatal and infant health outcomes: a systematic review and meta-analysis.[J]. Paediatric and perinatal epidemiology, 2012, 26 Suppl 1(0 1): 75-90.	Critical low quality
33.	Tripathi	2019	Tripathi P, Rao Y, Pandey K, et al. Significance of Vitamin D on the susceptibility of gestational diabetes mellitus - A meta-analysis[J]. Indian Journal of Endocrinology and Metabolism, 2019, 23(5): 514-524.	Critical low quality

34.	Wakoya	2023	Wakoya R, Afework M. Burden of Neural Tube Defects and Their Associated Factors in Africa: A Systematic Review and Meta-Analysis.[J]. International journal of pediatrics, 2023, 2023: 9635827.	Further findings do not meet the inclusion criteria
35.	Walker	2023	Walker K C, Thorsteinsdottir F, Christesen H T, et al. Vitamin D Supplementation and Vitamin D Status during Pregnancy and the Risk of Congenital Anomalies—A Systematic Review and Meta-Analysis[J]. Nutrients, 2023, 15(9).	Further findings do not meet the inclusion criteria
36.	Wang	2021	Wang L, Hou Y, Meng D, et al. Vitamin B12 and Folate Levels During Pregnancy and Risk of Gestational Diabetes Mellitus: A Systematic Review and Meta-Analysis.[J]. Frontiers in nutrition, 2021, 8: 670289.	Further findings do not meet the inclusion criteria
37.	Wang	2023	Wang S, Shi M, Zhou L, et al. Correlation of vitamin E level during pregnancy with maternal and neonatal health outcomes: a meta-analysis and systematic review[J]. American Journal of Translational Research, 2023, 15(6): 3838-3845.	Critical low quality
38.	Wang	2020	Wang L, Zhang C, Song Y, et al. Serum vitamin D deficiency and risk of gestational diabetes mellitus: A meta-analysis[J]. Archives of Medical Science, 2020, 16(4): 742-751.	Critical low quality
39.	Yu	2023	Yu M, Shen Q. Effects of maternal folic acid supplementation on renal urinary system development in human offspring—a meta-analysis and systemic review[J]. Pediatric Medicine, 2023, 6.	Further findings do not meet the inclusion criteria
40.	Zhang	2017	Zhang H, Huang Z, Xiao L, et al. Meta-analysis of the effect of the maternal vitamin D level on the risk of spontaneous pregnancy loss[J]. International Journal of Gynecology and Obstetrics, 2017, 138(3): 242-249.	Critical low quality
41.	Zhao	2022	Zhao R, Zhou L, Wang S, et al. Association between maternal vitamin D levels and risk of adverse pregnancy outcomes: a systematic review and dose-response meta-analysis.[J]. Food & function, 2022, 13(1): 14-37.	Further findings do not meet the inclusion criteria

42.	Zhao	2022	Zhao R, Zhou L, Wang S, et al. Effect of maternal vitamin D status on risk of adverse birth outcomes: a systematic review and dose–response meta-analysis of observational studies[J]. European Journal of Nutrition, 2022, 61(6): 2881-2907.	Further findings do not meet the inclusion criteria
43.	Zhou	2017	Zhou S S, Tao Y H, Huang K, et al. Vitamin D and risk of preterm birth: Up-to-date meta-analysis of randomized controlled trials and observational studies[J]. Journal of Obstetrics and Gynaecology Research, 2017, 43(2): 247-256.	Study overlap

Figure S1 Forest plots of the patients with GDM vs healthy controls

Study	Experimental	Control	WMD	95%CI	Weight
Arnold 2015	135	517	-6.90	(-11.26, -2.54)	2.3%
Akhlaghi 2014	61	69	-5.70	(-7.47, -3.92)	14.1%
Baker 2011	60	120	11.00	(2.67, 19.33)	0.6%
Clifton-Bligh 2008	81	226	-6.70	(-12.92, -0.48)	1.1%
Farrant 2009	39	520	2.90	(-7.92, 13.72)	0.4%
Haidari 2016	45	45	-8.16	(-15.23, -1.1)	0.9%
Hosseini 2018	82	82	-8.79	(-15.85, -1.72)	0.9%
Lacroix 2014	54	601	-6.00	(-10.83, -1.17)	1.9%
Maghbooli 2008	52	527	-6.48	(-10.87, -2.09)	2.3%
Makgoba 2011	90	158	-0.40	(-7.31, 6.51)	0.9%
McManus 2014	36	37	-15.90	(-25.96, -5.84)	0.4%
Mostali 2018	48	48	-9.50	(-15.9, -3.1)	1.1%
Parildar 2013	44	78	-8.49	(-17.31, 0.33)	0.6%
Park 2014	23	500	1.40	(-6.82, 9.62)	0.7%
Parlea 2012	118	219	-5.70	(-10.22, -1.18)	2.2%
Pleskacova 2015	47	29	-3.85	(-4.81, -2.89)	48.1%
Sobhani 2016	77	77	-8.93	(-16.3, -1.57)	0.8%
Soheilykhah 2010	54	111	-3.28	(-5.84, -0.72)	6.8%
Wang 2012	200	200	-3.50	(-5.76, -1.24)	8.7%
Zhang CL 2008	57	114	-14.73	(-21.81, -7.65)	0.9%
Zuhur 2013	234	168	-5.20	(-8.42, -1.98)	4.3%
**Overall**	**1637**	**4446**	**-4.52**	**(-5.19, -3.85)**	

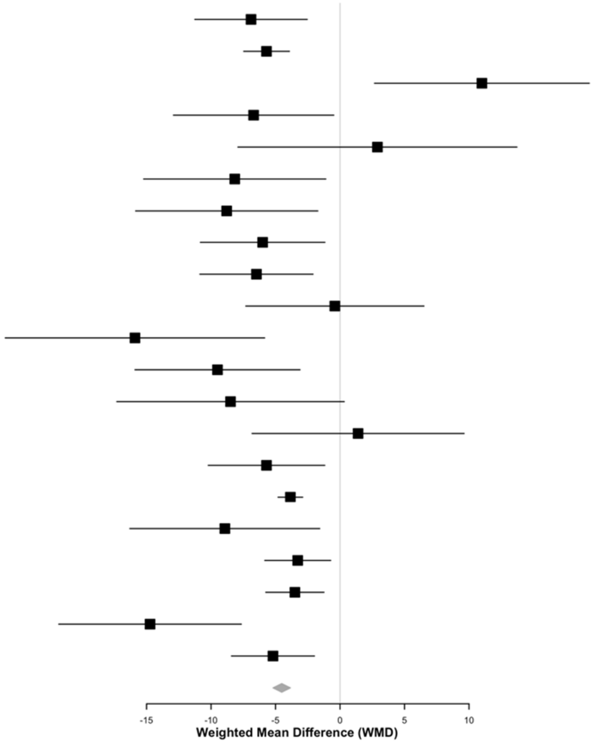

Figure S2 Funnel plot to access the publication bias

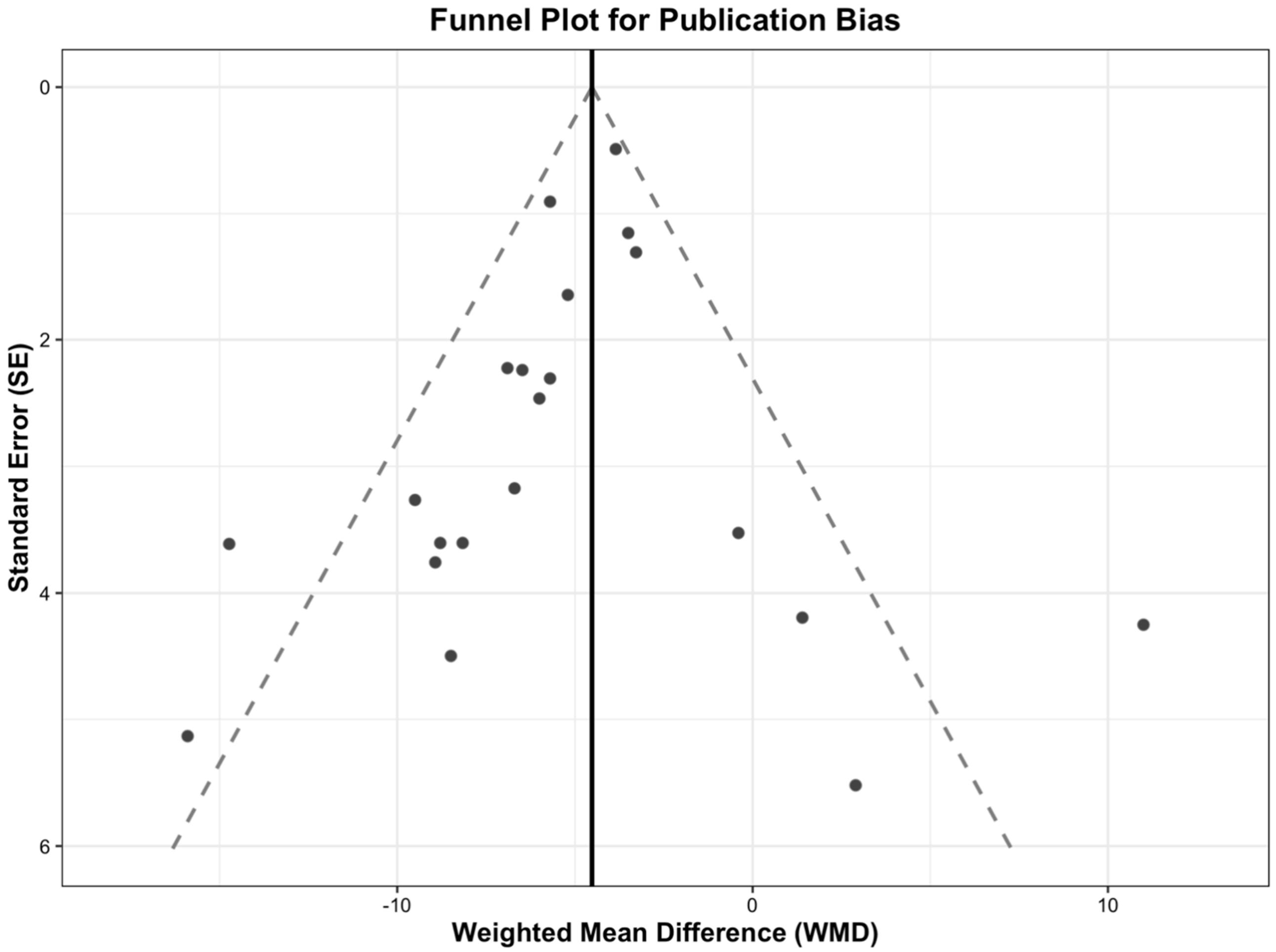

Supplement: Supplementary file 1 [file jcm-15-08434-s001.zip › Supplementary materials.pdf]
